# Carpel-specific down-regulation of *GhCKXs* in cotton significantly enhances seed and fiber yield

**DOI:** 10.1093/jxb/erac303

**Published:** 2022-07-06

**Authors:** Jianyan Zeng, Xingying Yan, Wenqin Bai, Mi Zhang, Yang Chen, Xianbi Li, Lei Hou, Juan Zhao, Xiaoyan Ding, Ruochen Liu, Fanlong Wang, Hui Ren, Jingyi Zhang, Bo Ding, Haoru Liu, Yuehua Xiao, Yan Pei

**Affiliations:** Biotechnology Research Center, Southwest University, Beibei, Chongqing, P. R. China; Biotechnology Research Center, Southwest University, Beibei, Chongqing, P. R. China; Biotechnology Research Center, Southwest University, Beibei, Chongqing, P. R. China; Biotechnology Research Center, Southwest University, Beibei, Chongqing, P. R. China; Biotechnology Research Center, Southwest University, Beibei, Chongqing, P. R. China; Biotechnology Research Center, Southwest University, Beibei, Chongqing, P. R. China; Biotechnology Research Center, Southwest University, Beibei, Chongqing, P. R. China; Biotechnology Research Center, Southwest University, Beibei, Chongqing, P. R. China; Biotechnology Research Center, Southwest University, Beibei, Chongqing, P. R. China; Biotechnology Research Center, Southwest University, Beibei, Chongqing, P. R. China; Biotechnology Research Center, Southwest University, Beibei, Chongqing, P. R. China; Biotechnology Research Center, Southwest University, Beibei, Chongqing, P. R. China; Biotechnology Research Center, Southwest University, Beibei, Chongqing, P. R. China; Biotechnology Research Center, Southwest University, Beibei, Chongqing, P. R. China; Biotechnology Research Center, Southwest University, Beibei, Chongqing, P. R. China; Biotechnology Research Center, Southwest University, Beibei, Chongqing, P. R. China; Biotechnology Research Center, Southwest University, Beibei, Chongqing, P. R. China; University of Nottingham, UK

**Keywords:** AG subfamily gene, carpel-specific, cotton yield, cytokinin, cytokinin oxidase, ovule initiation

## Abstract

Cytokinin is considered to be an important driver of seed yield. To increase the yield of cotton while avoiding the negative consequences caused by constitutive overproduction of cytokinin, we down-regulated specifically the carpel genes for cytokinin oxidase/dehydrogenase (CKX), a key negative regulator of cytokinin levels, in transgenic cotton. The carpel-specific down-regulation of *CKX*s significantly enhanced cytokinin levels in the carpels. The elevated cytokinin promoted the expression of carpel- and ovule-development-associated genes, *GhSTK2*, *GhAG1*, and *GhSHP*, boosting ovule formation and thus producing more seeds in the ovary. Field experiments showed that the carpel-specific increase of cytokinin significantly increased both seed yield and fiber yield of cotton, without resulting in detrimental phenotypes. Our study details the regulatory mechanism of cytokinin signaling for seed development, and provides an effective and feasible strategy for yield improvement of seed crops.

## Teaser

Carpel-specific enhancement of cytokinin significantly increases cotton ovule number and cotton yield without detrimental phenotypes.

## Introduction

Cotton is a major source of natural fibers for the global textile industry. Cotton seeds contain about 23% protein and 21% oil, and therefore are also an important source of foodstuff, feed, and edible oil (Zhang *et al.*, 2002; Sunilkumar *et al.*, 2006; Chen *et al.*, 2007; Ma *et al.*, 2016). A cotton boll contains approximately 30 seeds, and approximately 25% of the ovular epidermal cells of each ovule are able to differentiate into the commercially important lint fibers (Kim and Triplett, 2001; Mansoor and Paterson, 2012). Hence, the seed size and seed number of cotton determine the yield of fibers and seeds. However, seed size is negatively correlated with seed number due to the limitation of space and nutrition, and enhancing fiber abundance usually results in smaller seeds (J. Zhang *et al.*, 2005; M. Zhang *et al.*, 2011).

Generally, a seed is derived from a fertilized ovule, and thus, ovule development is critical to seed yield. The number of ovules per ovary depends on the ovule identity and ovule primordia initiation, which are controlled by an array of genes (Franks *et al.*, 2002; Favaro *et al.*, 2003; Sridhar *et al.*, 2006; Azhakanandam *et al.*, 2008; Jiang *et al.*, 2020), hormone signals (Higuchi *et al.*, 2004; Bartrina *et al.*, 2011; Bencivenga *et al.*, 2012; Zu *et al.*, 2022), and environmental factors (Meyer, 1966; Reddy *et al.*, 1991; Ritchie *et al.*, 2007; Sita *et al.*, 2017; Jiang *et al.*, 2019). Ovules originally arise from carpel (ovary) tissue as new meristematic formation, and the cell fate in proliferating ovule primordia is specified by particular ovule identity factors, such as MADS box AG subfamily members Seedstick (STK), Shatterproof (SHP1/2), and Agamous (AG) (Favaro *et al.*, 2003; Pinyopich *et al.*, 2003; Brambilla *et al.*, 2007; Zu *et al.*, 2022). It was reported that ectopic expression of their genes can promote the formation of carpels and ovules (Favaro *et al.*, 2003; Pinyopich *et al.*, 2003; Xu *et al.*, 2004; Guo *et al.*, 2007; Liu *et al.*, 2009, 2010; de Moura *et al.*, 2017; Nardeli *et al.*, 2018).

Cytokinins (CKs) are an important group of phytohormones that regulate the proliferation and differentiation of plant cells (Ioio *et al.*, 2008) and control many developmental and physiological processes in plants, including leaf senescence (Gan and Amasino, 1995; Hönig *et al.*, 2018), organ formation (Lohar *et al.*, 2004; Zhao *et al.*, 2009), nutrient uptake and allocation (Séguéla *et al.*, 2008; Gu *et al.*, 2018), as well as biotic and abiotic stress (Siemens *et al.*, 2006; Rivero *et al.*, 2007; Choi *et al.*, 2010; Peleg *et al.*, 2011; Cortleven *et al.*, 2019). A noteworthy role of cytokinins is to regulate seed yield (Sawan *et al.*, 2000; Ashikari *et al.*, 2005; Bartrina *et al.*, 2011).

The signal transduction pathway of cytokinins has been well investigated in Arabidopsis. The signaling pathway is initiated by binding of cytokinin to Arabidopsis histidine kinase receptors (AHKs), following phosphoryl group transport via Arabidopsis histidine proteins (AHPs) to Arabidopsis response regulators (ARRs). The B-type ARRs that contain conserved GARP DNA binding and activation domains can bind to a short 5ʹ-(A/G)GAT(T/C)-3ʹ core DNA sequence to activate the transcription of downstream genes (Sakai *et al.*, 2000, 2001; Mason *et al.*, 2004, 2005; Argyros *et al.*, 2008; Werner and Schmülling, 2009; Argueso *et al.*, 2010; Zubo and Schaller, 2020). Among ARR-activated genes, members of the AG subfamily, such as *STK*, *AG1*, and *SHP*, have been known to be involved in ovule initiation (Bartrina *et al.*, 2011; Zu *et al.*, 2022). However, details about cytokinin signaling pathway in carpel development need to be investigated.

Cytokinin levels are regulated through biosynthesis, activation, degradation, and conjugation of the bioactive molecules (Sakakibara, 2006; Jameson and Song, 2016). Cytokinin oxidase/dehydrogenase (CKX), which catalyses the irreversible degradation of the cytokinins by oxidative side chain cleavage, is a crucial regulator controlling endogenous cytokinin levels in the plant kingdom (Jones and Schreiber, 1997; Schmülling *et al.*, 2003; Kowalska *et al.*, 2010). Down-regulation of *CKX* genes could significantly increase cytokinin levels in transgenic plants and contribute to the enhancement of seed (grain) yield (Ashikari *et al.*, 2005; Zalewski *et al.*, 2012; Yeh *et al.*, 2015; Holubová *et al.*, 2018). However, constitutive down-regulation of *CKX*s in plants usually causes cytokinin overproduction phenotypes, including dwarfism, sterility, and root growth inhibition (Zalewski *et al.*, 2012; Gao *et al.*, 2014; Zhao *et al.*, 2015; Gasparis *et al.*, 2019). Thus, the key to regulating *CKX*s for seed yield improvement is to express the genes at the right time in the right place (Daskalova *et al.*, 2007; Werner *et al.*, 2010; Gao *et al.*, 2014; Ramireddy *et al.*, 2018).

In this study, we used a chimeric carpel- and stamen-specific promoter (Busch *et al.*, 1999; Deyholos and Sieburth, 2000), *proAGIP*, to down-regulate a functional cotton *CKX* gene (*proAGIP::GhCKX3b*-RNAi) in cotton carpels. We showed that the carpel-specific down-regulation of *GhCKX*s could enhance CK levels at the position where the ovule initiates in the carpel, without causing abnormal growth phenotypes. Cytokinin-activated GhARR1, GhARR2a, and GhARR11 in turn promoted the expression of *GhSTK2*, *GhAG1*, and *GhSHP* by binding their promoter sequences, thus boosting carpel development and ovule formation. As a result, seed and fiber yield of *proAGIP::GhCKX3b*-RNAi cotton was significantly increased compared with that of the non-transgenic control. Our results indicate the biotechnological potential of manipulation of CKs in cotton carpels for the concurrent improvement of seed and fiber yield.

## Materials and methods

### Plasmid construction and plant materials

The construction of *GhCKX3b*-RNAi (previously named *GhCKX*-RNAi) and the genotype of *pro35S::GhCKX*-RNAi transgenic cotton have been described previously (Zeng *et al.*, 2012; Zhao *et al.*, 2015). To construct the vectors used for genetic transformation, the 1653 bp sequence of the 3ʹ end of the second intron of the *AtAG* gene was amplified from the gDNA of Arabidopsis and fused with a 46 bp minimal CaMV35S promoter to create a functional *proAGIP* promoter, as previously described (Fang *et al.*, 1989; Deyholos and Sieburth, 2000). For the *proAGIP::GhCKX3b*-RNAi construct, the *proAGIP* promoter was linked with the *GhCKX3b*-RNAi fragment and assembled with *Hin*dIII and *Eco*RI-linearized p5 vector (Luo *et al.*, 2007). *proAGIP::GUS* was constructed by replacing the CaMV35S promoter of the pBI121 vector with the *proAGIP*. To construct the vectors used for dual-luciferase and protein subcellular localization assays, the coding regions of *GhARR1*, *GhARR2a*, *GhARR2b*, *GhARR11*, and *GhARR12* were amplified from carpel cDNA of upland cotton ‘Jimian 14’ (with or without stop codon). Similarly, *Yellow Fluorescent Protein* (*YFP*) was amplified from plasmid pLGN-*pro35S::GhPIN3a::YFP* (Zeng *et al.*, 2019) (with or without stop codon). For the *pro35S::GhARR1*, *pro35S::GhARR2a*, *pro35S::GhARR2b*, *pro35S::GhARR11*, and *pro35S::GhARR12* constructs, the coding regions of *GhARR1*, *GhARR2a*, *GhARR2b*, *GhARR11*, and *GhARR12* (with stop codon), respectively, were assembled with *Spe*I and *Sal*I-linearized pLGN vector (Zeng *et al.*, 2019). For the *pro35S::GhARR1::YFP*, *pro35S::GhARR2a::YFP*, and *pro35S:GhARR2b::YFP* constructs, the coding regions of *GhARR1*, *GhARR2a*, and *GhARR2b* (without stop codon), respectively, were fused to the 5ʹ terminal region of *YFP* (with stop codon) and assembled with *Spe*I and *Eco*RI-linearized pLGN vector. For the *pro35S::YFP::GhARR11* and *pro35S::YFP::GhARR12* constructs, the coding regions of *GhARR11* and *GhARR12* (with stop codon), respectively, were fused to the 3ʹ terminal region of *YFP* (without stop codon) and assembled with *Spe*I and *Eco*RI-linearized pLGN vector. For the *proGhSTK1::LUC*, *proGhSTK2::LUC*, *proGhAG1::LUC*, and *proGhSHP::LUC* constructs, a 2998 bp upstream region from the start codon of *GhSTK1* (*proGhSTK1*, *−*2998 to −1 bp), a 2493 bp upstream region from the start codon of *GhSTK2* (*proGhSTK2*, *−*2493 to −1 bp), a 3000 bp upstream region from the start codon of *GhAG1* (*proGhAG1*, *−*3000 to −1 bp), and a 2998 bp upstream region from the start codon of *GhSHP* (*proGhSHP*, −2998 to −1 bp), respectively, were amplified from gDNA of upland cotton ‘Jimin 14’, and assembled with *Hin*dIII and *Bam*HI-linearized pGreenII 0800-LUC vector (Hellens *et al.*, 2005). For yeast one-hybrid (Y1H) assays, the partial sequences of *proGhSTK2* (−1491 to −476 bp), *proGhAG1* (−2425 to −1 bp), and *proGhSHP* (−2814 to −340 bp), respectively, were inserted into the pAbAi vector (Takara Bio, Japan) between sites *Kpn*I and *Xho*I, *Hin*dIII and *Xho*I, and *Kpn*I and *Xho*I to construct the pAbAi bait vectors. The coding regions of *GhARR1*, *GhARR2a*, and *GhARR11*, respectively, were inserted into the pGADT7 vector (Takara Bio, Japan) between sites *Nde*I and *Bam*HI, *Bam*HI and *Xho*I, and *Eco*RI and *Xho*I to construct the pGADT7 prey vectors. The constructs used for dual-luciferase and protein subcellular localization assays were transformed into *Agrobacterium tumefaciens* strain *GV3101*. The constructs used for Y1H assays were introduced into Y1H Gold Yeast (Takara Bio, Japan). The constructs used for genetic transformation were transformed into *Agrobacterium tumefaciens* strain LBA4404. Transgenic plants of tobacco (*Nicotiana tabacum*) and cotton ‘Jimian 14’ were generated using the method described before (Luo *et al.*, 2007), and grown in the green house at over 25 °C under a 16:8 h light–dark photoperiod or field condition. Gene specific primers used for plasmid construction are listed in Supplementary Table S1.

### RNA extraction and real-time quantitative PCR

Total RNA was extracted using the EASY spin plant RNA extraction kit (Aidlab Biotech, China). Approximately 1 μg RNA was transcribed into first-strand cDNA using the NovoScript Plus All-in-one First Strand cDNA Synthesis SuperMix (gDNA Purge, Novoprotein, China). The real-time quantitative PCR (RT-qPCR) assays were performed on a CFX Connect Real-Time System (Bio-Rad Laboratories) with 2×NovoStart SYBR qPCR SuperMix plus (Novoprotein, China). *GhHis3* (Zhang *et al.*, 2011; Wan *et al.*, 2016; Wu *et al.*, 2018) and *GhUbiquitin* (Walford *et al.*, 2011; Wu *et al.*, 2018) served as internal references. Gene specific primers used for RT-qPCR are listed in Supplementary Table S2. The expression data were calculated with the ΔΔ*C*_t_ method. For the RT-qPCR analysis, three individual biological replicates with two technical replicates for each gene were used. Mean values and standard errors were calculated using the data from the three replicates. The compliance of the RT-qPCR experiments with the Minimum Information for Publication of Quantitative Real-Time PCR Experiments (MIQE) is shown in a MIQE checklist (Supplementary Table S3).

### Sequence retrieval, phylogenetic analysis, and sequence alignment

The amino acid sequences of AtCKXs and B-type AtARRs were obtained from the Arabidopsis genome databases (https://www.Arabidopsis.org/, accessed on 30 December 2019). The GhCKX and B-type GhARR homologs were identified using the BLASTP tool with default parameters in the CottonFGD database (https://cottonfgd.org/, accessed on 30 December 2019) (Zhu *et al.*, 2017) using AtCKX and B-type AtARR sequences, respectively. The cutoff values were 0 for the sequence retrieval of GhCKXs and 1.0^−140^ for the sequence retrieval of B-type GhARRs. Then, the selected GhCKX sequences were used for further identification of GrCKXs and GaCKXs by searching the databases https://phytozome.jgi.doe.gov/pz/portal.html and https://cottonfgd.org/, respectively. The phylogenetic trees of deduced CKX and B-type ARR amino acids were constructed by the neighbor-joining algorithm with default parameters, with 1000 bootstrap replicates as implemented in MEGA5.0 software. The *GhCKX* sequences were aligned using Megalign DNAstar software (Burland, 2000) and analysed with the Gendoc software (Nicholas, 1997).

### Histochemical staining and quantification of β-glucuronidase activity

Histochemical staining of β-glucuronidase (GUS) was performed as previously described by Jefferson *et al.* (1987). Briefly, detached or hand-sectioned tissues were immediately immersed in the staining solution (Zeng *et al.*, 2019) and then placed in the dark at 37 °C for 12 h. The stained samples were bleached and fixed in 75% ethanol before photographing. Images were captured on a stereo-microscope imaging system (SteREO Discovery V20, Zeiss, Germany). Fluorometric assays of GUS activity in vegetative and reproductive organs of *proTCS::GUS* transgenic cotton were performed as described by Hou *et al.* (2008). Each sample was ground in liquid nitrogen. Protein estimation was performed using the method of Bradford (1976). GUS activity was calculated as pmol 4-methylumbelliferone (4-MU) per minute per microgram protein and each test was represented by three biological replicates.

### *In situ* hybridization

The linearized DNA template of the gene-specific *GhCKX3b* probe was amplified directly from a vector carrying the coding sequence of *GhCKX3b*. *In situ* hybridization of *GhCKX3*b mRNA was performed following the method described in Zhang *et al.* (2017). The sections incubated with the sense RNA probe served as the negative control. Images were captured on a microscope (CKX41, Olympus, Japan). Gene specific primers are listed in Supplementary Table S1.

### Quantification of endogenous cytokinin

Buds of cotton were harvested at the pinhead square stage (approximately −21 d post-anthesis; DPA), and then carpels (100 mg fresh weight) were separated and ground in liquid nitrogen. Extraction and detection of endogenous cytokinin was performed following the method described in Yoshimoto *et al.* (2009) and Zeng *et al.* (2019). Analytical parameters of LC-MS/MS are listed in Supplementary Table S4.

### Immunohistochemical localization of cytokinins

Immunolocalization of cytokinins was performed following the method described by Zhang *et al.* (2017) with some modifications. Briefly, sections (10 μm) of cotton bud at the pinhead square stage were incubated with antibody (against *trans*-zeatin riboside (tZR) and *trans*-zeatin (tZ), Agrisera, Sweden) and then the signal was detected by using DyLight 550-labeled secondary antibody (Abcam, UK), and visualized on a laser-scanning confocal microscope (SP8, Leica, Germany). Sections incubated without the primary antibody served as the negative control.

### Transient expression

Four-week-old leaves of *Nicotiana benthamiana* were used for transient expression. *Agrobacterium tumefaciens* strain GV3101 containing a plant expression vector was cultured overnight at 28 °C to OD_600_ of 1.0. The pelleted cells were resuspended and diluted with infiltration buffer (Chen *et al.*, 2021) to OD_600_ of 0.01–0.05. The infiltrated leaves were used for analysis 3 d later.

### Microscopic observations

GUS-stained samples were observed using a stereo-microscope imaging system (SteREO Discovery V20, Zeiss, Germany). Fiber initiation was observed on an S-3400N scanning electron microscope (Hitachi, Japan) as described previously by Zeng *et al.* (2019). The protein subcellular localization and cytokinin immunolocalization was observed on a laser-scanning confocal microscope (SP8, Leica, Germany). The fluorescence signal was detected by an HyD detector under a ×40 oil immersion objective lens. The imaging condition was set up manually: DyLight 550 (excitation: 552 nm, emission: 560–600 nm) and YFP (excitation: 514 nm, emission: 520–560 nm). The intensity of fluorescence signal was quantified using software Leica Application Suite X.

### Southern blot

Southern blot was conducted as previously described by Zhang *et al.* (2011). Briefly, gDNA of *proAGIP::GhCKX3b*-RNAi transformants and the non-transgenic control cotton was digested overnight (10 µg) with the restriction enzyme *Eco*RI. An *NPTII* fragment amplified from the vector p5 served as the probe. DIG High Prime DNA Labeling and Detection Starter Kit II (Roche, Germany) were employed to prepare digoxigenin-labeled probes and to detect hybridization signals. Gene specific primers are listed in Supplementary Table S1.

### Yeast one-hybrid assay

Y1H assays were performed as previously described by Li *et al.* (2021). In brief, the pAbAi bait vector was transformed into the yeast strain Y1H Gold by the LiAc-mediated method. The pGADT7 prey vector was transformed into Y1H Gold cells harboring the Bait-pAbAi. Transformants were screened in the synthetic dextrose medium (SDM) containing 100 or 200 ng ml^−1^ aureobasidin A (AbA) and lacking Ura and Leu (SDM/+AbA/−Leu). The yeast growth was captured after 5 d inoculation at 30 °C.

### Dual-luciferase reporter assay

A dual-luciferase reporter assay was performed as previously described by Hellens *et al.* (2005) and Yan *et al.* (2018). Briefly, the promoter fragments were amplified and ligated into the pGreenII 0800*-*LUC vector to produce firefly luciferase (LUC) reporters, and the coding regions of GhARRs were amplified and ligated into the pLGN vector to produce effectors. The reporters and effectors were co-infiltrated into 4-week-old leaves of *N. benthamiana* as described in ‘Transient expression’. The effector expressing *pro35S::YFP* served as internal control. After 3 d of growth at 25 °C, 10 μM tZ was infiltrated into the leaves 5 h before sampling. The leaves were infiltrated with the same volume of dimethyl sulfoxide (DMSO) as the negative control (0 μM tZ). The Dual-Glo Luciferase Assay System (Promega, USA) was employed to measure the LUC activity, which was calculated based on the ratio of LUC/*Renilla* luciferase (REN). Gene specific primers are listed in Supplementary Table S1.

### Statistical analysis

Statistical analysis was performed with Student’s *t*-test or one-way ANOVA followed by Tukey multiple comparisons test (*P*<0.05). Each experiment comprised at least three replicates. The intensity of fluorescence signal was calculated using Leica Application Suite X software. Standard errors and standard deviations were calculated using Microsoft Excel (2016) and IBM SPSS Statistics (version 19).

## Results

### *GhCKX3b* is preferentially expressed in carpels

**Fig. 1.**
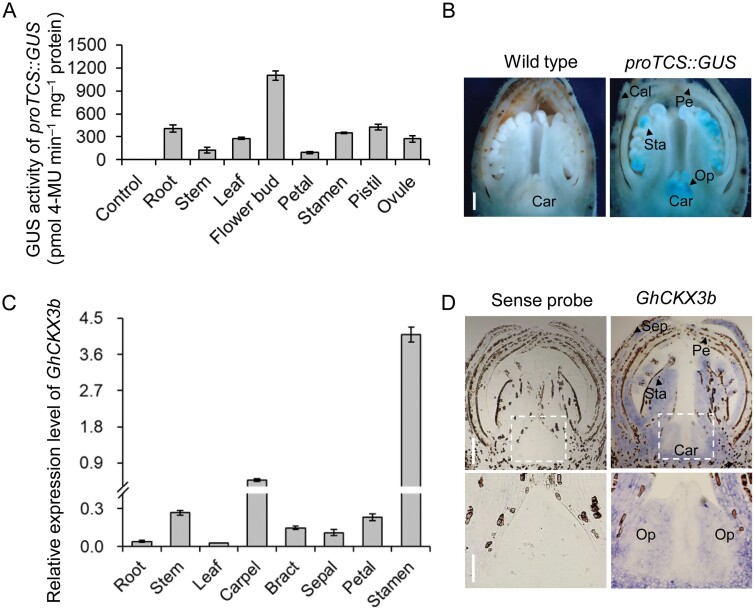
Cytokinin activities and *GhCKX3b* transcription levels in different cotton tissues. (A, B) The endogenous cytokinin activities indicated by GUS activities (A) and GUS staining (B) of the proTCS::GUS reporter system in cotton tissues. GUS activities were measured by methylumbelliferyl glucuronide assay in *proTCS::GUS* transgenic cotton roots (10 d after sowing; DAS), stems (the third internode from the apex at 110 DAS cotton plants), leaves (the third leaf from the apex), flower buds (approximately −21 DPA), petals, stamens, pistils, and ovules at anthesis. Wild-type flower buds were used as the negative control. Error bars indicate standard deviation (SD) of three biological repeats. Buds at approximately −21 DPA were used for GUS staining. (C, D) The *GhCKX3b* transcription levels detected by RT-qPCR (C) and mRNA *in situ* hybridization (D) in wild-type roots (10 DAS), stems (the third internode from the apex at 110 DAS cotton plants), leaves (the third leaf from the apex), carpels (ovaries), bracts, sepals petals, and stamens at approximately −21 DPA. *GhHis3* and *GhUbiquitin* served as internal control. Error bars indicate SD of three replicates. Bud sections of 10 µm at approximately −21 DPA were used for *in situ* hybridization with gene-specific probe *GhCKX3b*. The sections incubated with sense RNA probe served as the negative control. The lower panels in (D) show the enlarged image of carpels (ovaries). Scale bars=500 μm. Car, carpel; Op, ovule primordia; Pe, petal; Sep, sepal; Sta, stamen.

By detecting GUS activity, we estimated cytokinin activities in vegetative and reproductive organs of cotton expressing the cytokinin signaling reporter *proTCS::GUS* (Müller and Sheen, 2008; Zeng *et al.*, 2019). The maximum GUS activity was discernible in the flower bud at the pinhead square stage (approximately −21 DPA) when the ovule formation begins (Fig. 1A, B), suggesting that a high activity of cytokinins is required for ovule morphogenesis.

To identify cotton CKXs, which are the key negative regulators of cytokinins in plants (Schmülling *et al.*, 2003), we screened the genomic database of *Gossypium hirsutum* (https://cottonfgd.org/). Twenty-seven *GhCKX* homologous genes to Arabidopsis *AtCKXs* (https://www.arabidopsis.org/) were identified (Supplementary Fig. S1). Among them, *GhCKX3b*, *GhCKX3c*, *GhCKX5a*, and *GhCKX6b* were expressed preferentially in the carpel and stamen (Fig. 1C; Supplementary Fig. S2). Our previous study demonstrated that GhCKX3b is a functional cytokinin oxidase (previously named GhCKX; Zeng *et al.*, 2012; Zhao *et al.*, 2015). *In situ* mRNA hybridization confirmed a strong signal of *GhCKX3b* transcript in the carpel at the place where ovules formed (Fig. 1D).

### Specific down-regulation of *GhCKX3b* increases CK levels in carpel

**Fig. 2.**
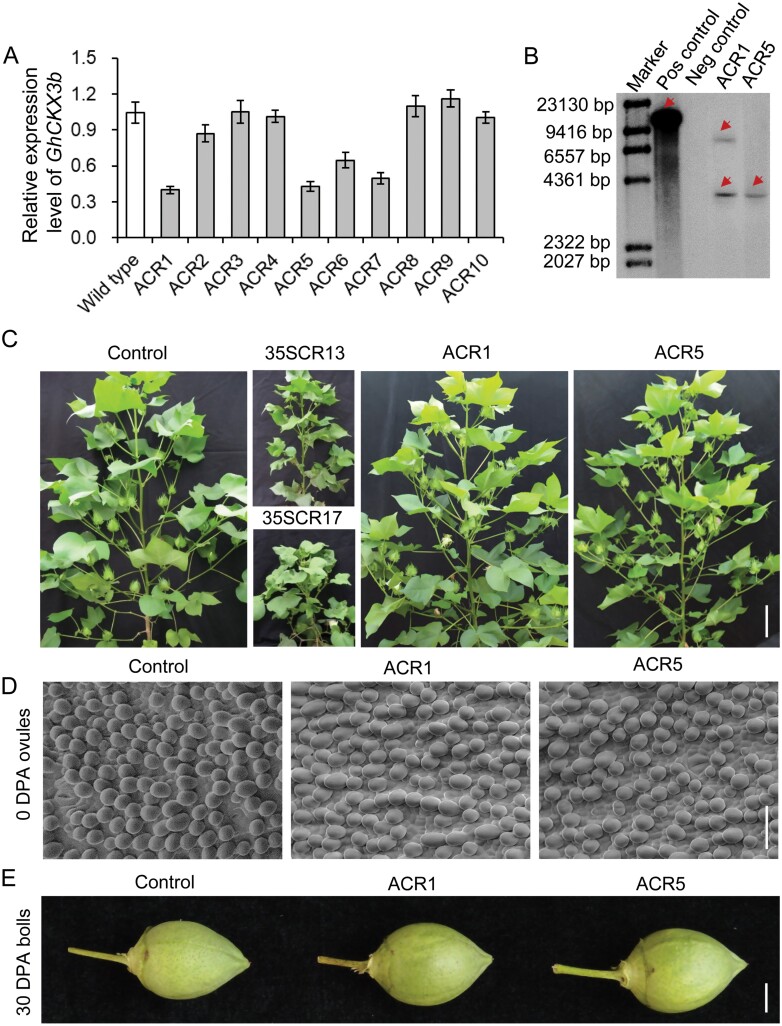
Molecular and phenotypic identification of *proAGIP::GhCKX3b*-RNAi transgenic cotton. (A) *GhCKX3b* transcription levels in cotton carpels (ovaries) at the pinhead square stage (approximately −21 DPA) of T_0_*proAGIP::GhCKX3b*-RNAi transgenic lines and wild type. *GhHis3* and *GhUbiquitin* served as internal control. Error bars indicate SD of three replicates. (B) Southern blot analysis of T_1_*proAGIP::GhCKX3b*-RNAi transgenic lines ACR1 and ACR5. Red arrows indicate the positive hybridization signal. (C) Phenotypes of 120 DAS cotton plants grown in the field. Control, non-transgenic segregated line of *proAGIP::GhCKX3b*-RNAi transgenic cotton; 35SCR13 and 35SCR17, lines #13 and #17 of *pro35S::GhCKX3b*-RNAi transgenic cotton; scale bar=10 cm. (D) Fiber initiation on 0 DPA ovules; scale bar=50 μm. (E) The phenotype of 30 DPA cotton bolls; scale bar=1 cm.

Down-regulation of *CKX*s could significantly increase cytokinin levels in transgenic plants (Ashikari *et al.*, 2005; Zalewski *et al.*, 2012; Yeh *et al.*, 2015; Holubová *et al.*, 2018). However, constitutional down-regulation of *CKXs* in cotton usually resulted in cytokinin overproduction phenotypes, including dwarfism, sterility, and shortened root (Fig. 2C; Zhao *et al.*, 2015).

To avoid the side effects on plant growth from the global overproduction of cytokinins, while increasing the seed number through cytokinin manipulation, we decided to increase the cytokinin content spatiotemporally in carpels. To this end, we used *proAGIP*, a chimeric carpel- and stamen-specific promoter (Busch *et al.*, 1999; Deyholos and Sieburth, 2000), to direct the transcription of a *GhCKX3b*-RNAi sequence (Supplementary Fig. S3). The GUS pattern in *proAGIP::GUS* transgenic tobacco (*Nicotiana tabacum*) confirmed the carpel-specificity of *proAGIP* promoter (Supplementary Fig. S4; Deyholos and Sieburth, 2000; Wang *et al.*, 2008).

**Fig. 3.**
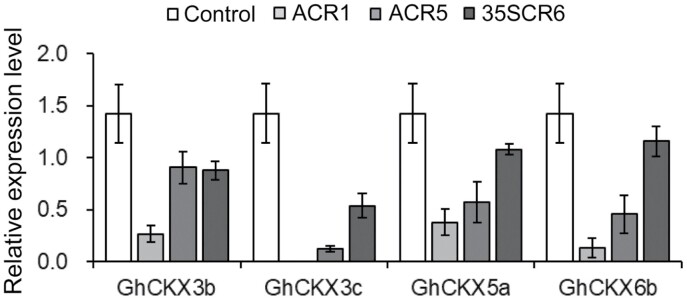
*GhCKXs* transcription levels in cotton carpels at the pinhead square stage. Total RNAs were extracted from cotton carpels at the pinhead square stage (approximately −21 DPA). The relative transcription levels were determined by RT-qPCR. *GhHis3* and *GhUbiquitin* served as internal control. Error bars indicate SD of three replicates. Control, non-transgenic segregated line of *proAGIP::GhCKX3b*-RNAi transgenic cotton. ACR, T_2_*proAGIP::GhCKX3b*-RNAi transgenic cotton. 35SCR6, line #6 of T_5_*pro35S::GhCKX3b*-RNAi transgenic cotton.

No discernable phenotypic alteration in plant growth and development was observed in transgenic cotton holding *proAGIP::GhCKX3b*-RNAi (ACR) (Fig. 2C–E). RT-qPCR showed that the transcriptional level of *GhCKX3b* in the carpel of transgenic lines was obviously reduced. Two transgenic lines, ACR1 and ACR5, in which *GhCKX3b* was largely down-regulated, were selected for further study (Figs 2A, B, 3).

**Fig. 4.**
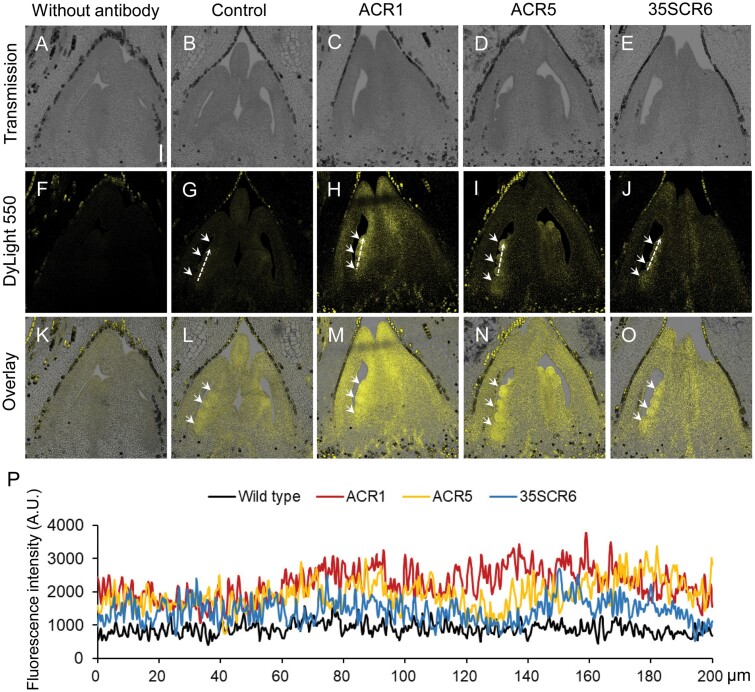
Immunolocalization of active CKs in carpels at the pinhead square stage. (A–O) Carpels at the pinhead square stage (approximately −21 DPA) were used for the immunolocalization of CKs. Bud sections of 10 µm were incubated with the antibody (against tZR and tZ) and then the signal was detected by a secondary antibody conjugated to DyLight 550. Sections incubated without the primary antibody served as the negative control. Arrows indicate where ovules formed. Scale bar=100 μm. (P) Fluorescence intensity along the white dashed arrows (in G–J, respectively). The fluorescence intensity is given in arbitrary units (A.U.). Control, non-transgenic segregated line of *proAGIP::GhCKX3b*-RNAi transgenic cotton; ACR, T_2_*proAGIP::GhCKX3b*-RNAi transgenic cotton; 35SCR6, line #6 of T_5_*pro35S::GhCKX3b*-RNAi transgenic cotton.

Then, we performed LC-MS/MS to detect the content of six primarily active cytokinins, namely tZ, tZR, N6-isopentenyladenine, N6-isopentenyladenosine, dihydrozeatin, and dihydrozeatin riboside, in carpels of cotton. Besides a non-transgenic negative control, a transgenic *pro35S::GhCKX*-RNAi line, 35SCR6, which showed a moderate increase of cytokinins with normal growth, and had the best yield performance among the *pro35S::GhCKX*-RNAi cottons (Zhao *et al.*, 2015), was used as a positive control. In carpels of ACR1 and ACR5 transgenic cotton, the total cytokinins were 29.53 ± 1.46 ng g^−1^ and 27.46 ± 1.63 ng g^−1^, respectively, significantly higher than in the 35SCR6 line (23.46 ± 1.81 ng g^−1^) and the non-transgenic control (15.05 ± 1.73 ng g^−1^) (Table 1), indicating a carpel-specific increase of cytokinins by *proAGIP::GhCKX3b*-RNAi. Immunolocalization assays supported that tZ and tZR signals, which made up the majority (85.0%) of the active CKs (Table 1), mainly appeared at the position where the ovule initiated, and the signals from ACR1 and ACR5 were visibly stronger than those in 35SCR6 and the control (Fig. 4).

**Table 1.** Active CK content in carpels during cotton ovule initiation (ng g^−1^ FW)

Line	tZ	tZR	iP	iPR	DZ	DZR	Total CKs
Control	5.17 ± 0.51	7.63 ± 0.78	0.47 ± 0.16	1.50 ± 0.21	0.19 ± 0.05	0.09 ± 0.01	15.05 ± 1.73 (c)
ACR1	10.04 ± 0.31	15.62 ± 0.84	0.90 ± 0.07	2.51 ± 0.22	0.28 ± 0.00	0.19 ± 0.01	29.53 ± 1.46 (a)
ACR5	10.68 ± 0.28	14.23 ± 0.15	0.56 ± 0.29	1.53 ± 0.80	0.28 ± 0.10	0.17 ± 0.01	27.46 ± 1.63 (a)
35SCR6	8.57 ± 0.43	12.20 ± 0.80	0.76 ± 0.13	1.58 ± 0.37	0.21 ± 0.07	0.14 ± 0.01	23.46 ± 1.81 (b)

Sampled carpels (ovaries) from buds at the pinhead square stage (approximately −21 DPA). Total CKs, sum of contents of tZ (*trans*-zeatin), tZR (tZ riboside), iP (*N*^6^-isopentenyladenine), iPR (*N*^6^-isopentenyladenosine), DZ (dihydrozeatin), and DZR (DZ riboside). Data are presented as means ±SD (*n*=3). Within each column, means that are followed different letters are significantly different at *P*<0.05 by one-way ANOVA with a Tukey multiple comparisons test. FW, fresh weight; Control, non-transgenic segregated line of *proAGIP::GhCKX3b*-RNAi transgenic cotton; ACR, *proAGIP::GhCKX3b*-RNAi transgenic cotton; 35SCR6, line #6 of *pro35S::GhCKX3b*-RNAi transgenic cotton.

### Increase of cytokinin promotes the expression of ovule initiation-related genes

**Fig. 5.**
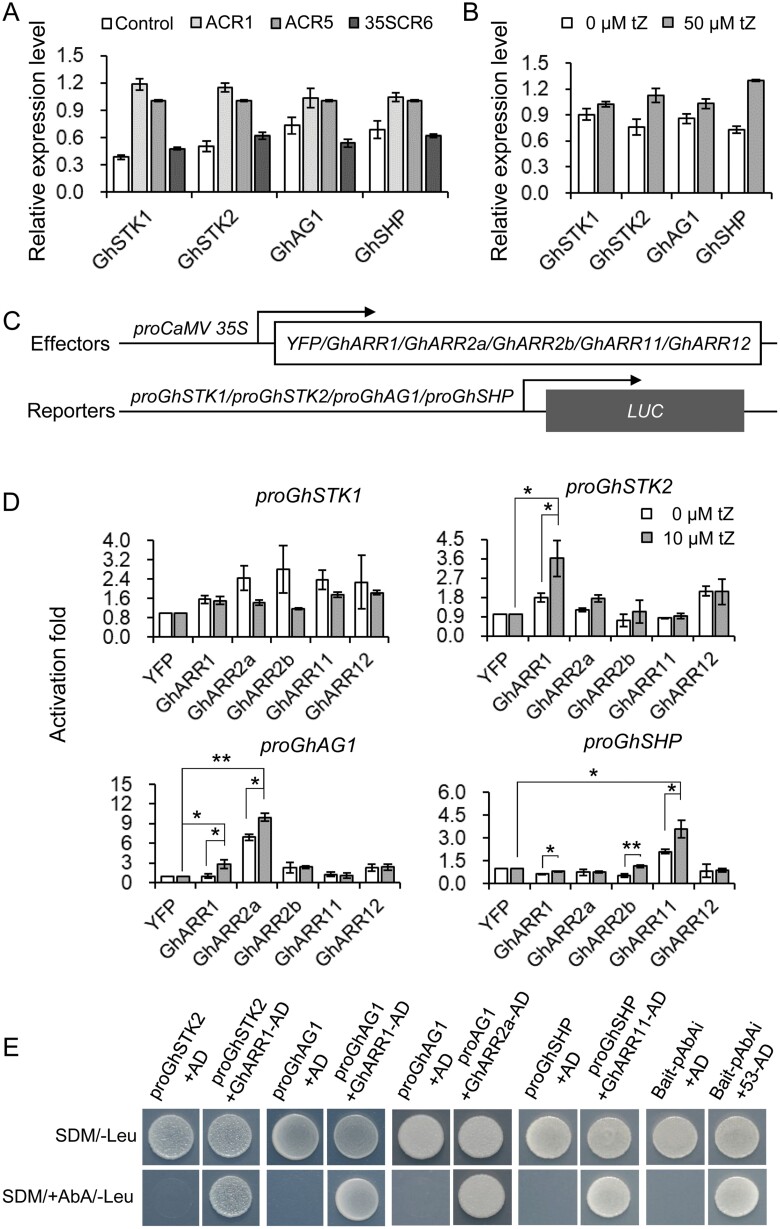
Increase of cytokinin promoted the expression of ovule initiation-related genes. (A) Comparison of the relative expression levels of AG subfamily genes in T_2_ ACR1 and ACR5 transgenic lines with those of the T_5_ 35SCR6 and the non-transgenic control. Total RNAs were extracted from cotton carpels at the pinhead square stage (approximately −21 DPA). The relative transcription levels were determined by RT-qPCR. *GhHis3* and *GhUbiquitin* served as internal control. Error bars indicate SD of three replicates. Control, non-transgenic segregated line of *proAGIP::GhCKX3b*-RNAi transgenic cotton; ACR, *proAGIP::GhCKX3b*-RNAi transgenic cotton; 35SCR6, line #6 of *pro35S::GhCKX3b*-RNAi transgenic cotton. (B) The relative transcription levels of AG subfamily genes in tZ-treated ovules and the control. Total RNA was extracted from 0 DPA ovules treated with 50 μM tZ or the same volume of DMSO for the negative control, for 12 h. (C) Schematic representation of the constructs used in the dual-luciferase assay. (D) The promoters of AG subfamily genes involved in ovule initiation were activated under the expression of GhARRs and treatment with 10 μM tZ via a dual-luciferase assay. The effector expressing *pro35S::YFP* served as internal control. The leaves were infiltrated with 10 μM tZ or the same volume of DMSO for the negative control (0 μM tZ). The transactivation activity of AG subfamily gene promoters by GhARRs was calculated based on the ratio LUC/REN. Error bars indicate SD of three biological replicates. Asterisks represent significant difference (versus internal control, or 0 μM tZ) as determined by Student’s *t*-test (*P<0.05; **P<0.01). (E) Yeast one-hybrid assay of GhARRs binding to the promoters of AG subfamily genes. Promoter fragments were inserted into the pAbAi vector to construct pAbAi bait vectors, and the full-length coding sequences of *GhARRs* were inserted into the pGADT7 vector to construct pGADT7 prey vectors. The pGADT7 prey vector was transformed into Y1H Gold cells harboring the Bait-pAbAi. The Bait-pAbAi and 53-pGADT7 (53-AD) vectors served as the positive control. Possible interactions were screened in synthetic dextrose medium (SDM) containing 100 or 200 ng ml^−1^ aureobasidin A (AbA) and lacking Ura and Leu (SDM/+AbA/−Leu).

It has been reported that the expression of genes of the AG subfamily, such as those for Seedstick (STK), Shatterproof (SHP), and Agamous (AG), was able to promote the formation of carpels and ovules (Favaro *et al.*, 2003; Pinyopich *et al.*, 2003; Xu *et al.*, 2004; Guo *et al.*, 2007; Liu *et al.*, 2009, 2010; de Moura *et al.*, 2017; Nardeli *et al.*, 2018). In line with previous reports, AG subfamily genes, including *GhSTK1*, *GhSTK2*, *GhSHP*, and *GhAG1* were up-regulated in the carpel of the ACR1 and ACR5 lines (Fig. 5A; Supplementary Table S5). To confirm these results, we treated 0 DPA wild-type ovules with 50 μΜ tZ, an active cytokinin, for 12 h. The exposure resulted in a noticeable increase in the expression of AG subfamily genes (Fig. 5B), confirming the promotive effect of the cytokinin on the expression of these genes.

Cytokinins regulate the expression of downstream signal transduction genes by activating B-type ARRs (Hwang and Sheen, 2001; Meng *et al.*, 2017). To identify B-type ARR genes that are involved in cotton carpel development, we conducted a comparative phylogenetic analysis using data from Arabidopsis (https://www.arabidopsis.org/) and *Gossypium hirsutum* (https://cottonfgd.org/) (D’Agostino *et al.*, 2000; Sakai *et al.*, 2000; Mason *et al.*, 2004, 2005; Zubo and Schaller, 2020). Five carpel expressive GhARRs, i.e. GhARR1, GhARR2a, GhARR2b, GhARR11, and GhARR12, which predominantly localize to the nuclei of *N. benthamiana* pavement cells, were identified (Supplementary Fig. S5). Dual-luciferase reporter and Y1H assays indicated that GhARR1 could bind directly to the promoter sequences of the AG subfamily genes *GhSTK2* and *GhAG1*, GhARR2a to the promoter of *GhAG1*, and GhARR11 to the promoter of *GhSHP* (Fig. 5E; Supplementary Fig. S6). This binding could activate the transcription of these AG subfamily genes (Fig. 5C–D), thereby promoting the expression of AG downstream genes.

### Carpel-specific down-regulation of *GhCKXs* significantly increases seed and fiber yield

**Fig. 6.**
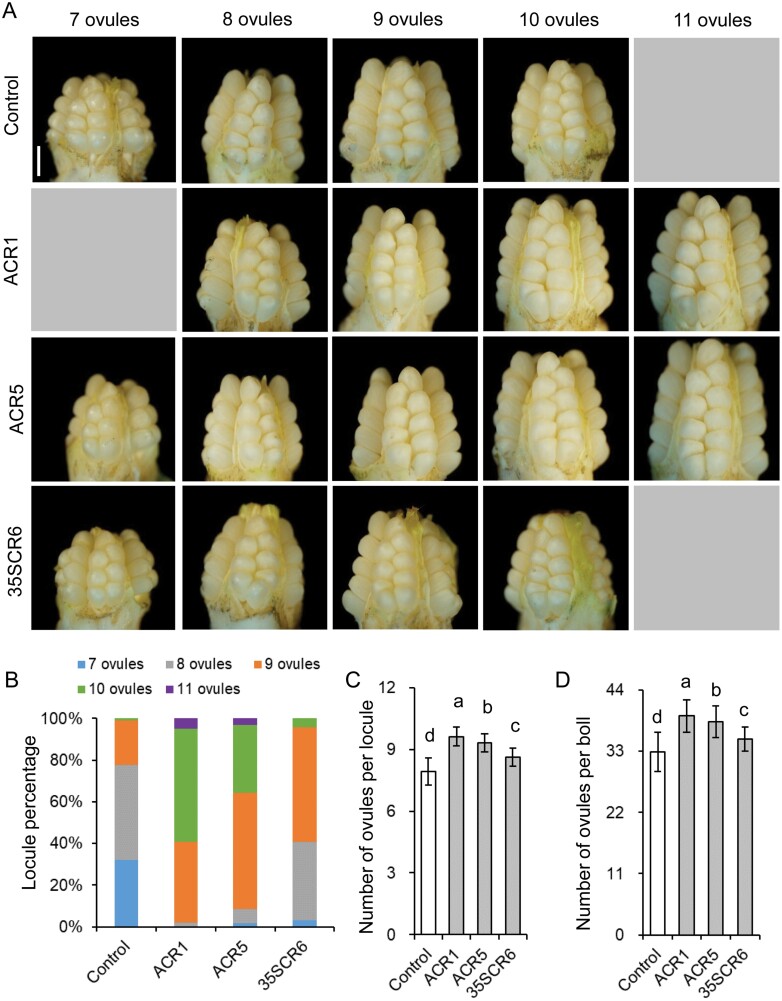
Comparison of ovule number in ACR1 and ACR5 transgenic lines with those of the 35SCR6 and the non-transgenic control. (A) Phenotypes of 0 DPA bolls peeled off shells. Bolls of T_2_ ACR1 and ACR5 and T_5_ 35SCR6, as well as the non-transgenic control at 0 DPA, were harvested and the shells were peeled off. (B) Locule percentage. The ratio represents the percentage of locules containing a different number of ovules. (C, D) Ovule number per locule or boll. Error bars indicate SD of 45 bolls in each experiment. Different letters in (C, D) represent significant differences at *P*<0.05 by one-way ANOVA followed by Tukey multiple comparisons test. Control, non-transgenic segregated line of *proAGIP::GhCKX3b*-RNAi transgenic cotton; ACR, *proAGIP::GhCKX3b*-RNAi transgenic cotton; 35SCR6, line #6 of *pro35S::GhCKX3b*-RNAi transgenic cotton.

By counting, we found that the number of ovules per locule (0 DPA) of ACR1 and ACR5 was significantly higher than that of the non-transgenic control as well as the constitutively down-regulated 35SCR6 line (Fig. 6; Supplementary Fig. S7). The average number of ovules per locule of the ACR1 and ACR5 lines was 9.6 ± 0.5 and 9.3 ± 0.4, respectively, increasing to 21.5% and 17.7%, respectively, over that of the non-transgenic control (7.9 ± 0.6), and 11.6% and 8.1%, respectively, over that of the 35SCR6 line (8.6 ± 0.4). For ACR1 locules, the majority (59.2%) contained 10 or 11 ovules in each locule. In contrast, for the non-transgenic cotton, the majority (77.5%) had seven or eight ovules, and only a small part (1.1%) had 10 ovules. For the 35SCR6 cotton, 4.3% of locules had 10 ovules, and none had 11 ovules. The increase of ovules per locule of ACR cotton was also constant during the flower stage of cotton development (Supplementary Fig. S8).

More ovules per locule usually means more seeds per boll. To assess the agronomic performance of the *proAGIP::GhCKX3b*-RNAi transgenic cotton, we conducted field trials at the experimental farm located in Chongqing, China (29°49ʹ11″N, 106°24ʹ43″E) in 2018 and 2019. The consecutive two-year field experiments showed a significant increase of seeds per boll in ACR lines (Table 2). The seed number per boll of ACR1 and ACR5 was 31.1 ± 0.7 and 29.8 ± 0.6, respectively, significantly higher than that of 35SCR6 (26.1 ± 0.9) and the control (24.8 ± 0.2). Consequently, the seed yield of ACR1 and ACR5 increased 22.4% and 26.5%, respectively, as compared with that of the control. The increased seeds resulted in a significant increase of lint yield. The lint yield of ACR1 and ACR5 was increased by 32.3% and 25.8%, respectively, compared with the control. The seed yield of the ACR1 line was lower (but not statistically significant) than that of 35SCR6 cotton, due to a slight decrease in seed size (indicated by seed index, the weight in grams of 100 seeds). Nevertheless, the lint yield of the ACR1 line was still significantly higher than that of the 35SCR6 line (Table 2), with the advantage of more seeds per boll. The fiber quality, fiber length, and fiber strength of ACR1 were lower than those of the control; but the values of ACR5 were not significantly different from those of the control (Supplementary Table S6; Supplementary Fig. S9).

**Table 2.** Comparison of yield-related traits among transgenic cottons and non-transgenic segregated lines in the field trial

Year	Line	Boll number per plant	Seed number per boll	Seed index (g)	Lint index (g)	Seed cotton weight per boll (g)	Seed yield (kg/plot)	Lint yield (kg/plot)
2018	Control	32.0 ± 0.2 (c)	24.8 ± 0.1 (b)	10.1 ± 0.1 (b)	6.3 ± 0.1 (a)	4.1 ± 0.1 (c)	4.8 ± 0.1 (b)	3.0 ± 0.0 (c)
	ACR1	33.3 ± 0.1 (b)	30.5 ± 0.4 (a)	9.5 ± 0.0 (c)	6.4 ± 0.1 (a)	4.9 ± 0.1 (a)	5.8 ± 0.1 (a)	3.9 ± 0.1 (a)
	ACR5	33.7 ± 0.2 (b)	29.7 ± 0.6 (a)	10.1 ± 0.2 (b)	6.4 ± 0.1 (a)	4.9 ± 0.1 (a)	6.1 ± 0.2 (a)	3.8 ± 0.1 (a)
	35SCR6	34.5 ± 0.1 (a)	25.3 ± 0.1 (b)	11.5 ± 0.2 (a)	6.7 ± 0.2 (a)	4.6 ± 0.1 (b)	6.0 ± 0.1 (a)	3.5 ± 0.1 (b)
2019	Control	32.7 ± 0.2 (b)	25.0 ± 0.3 (c)	10.3 ± 0.2 (b)^,^ (c)	6.4 ± 0.2 (a)	4.1 ± 0.1 (d)	5.0 ± 0.0 (b)	3.1 ± 0.1 (d)
	ACR1	33.9 ± 0.4 (b)	31.7 ± 0.1 (a)	9.6 ± 0.3 (c)	6.7 ± 0.1 (a)	5.2 ± 0.1 (a)	6.2 ± 0.2 (a)	4.3 ± 0.0 (a)
	ACR5	33.7 ± 0.1 (b)	29.9 ± 0.7 (a)	10.3 ± 0.3 (b)	6.5 ± 0.3 (a)	5.0 ± 0.1 (b)	6.2 ± 0.2 (a)	3.9 ± 0.0 (b)
	35SCR6	34.6 ± 0.3 (a)	26.8 ± 0.5 (b)	10.7 ± 0.2 (a)	6.6 ± 0.1 (a)	4.7 ± 0.1 (c)	6.0 ± 0.0 (a)	3.7 ± 0.0 (c)
Average over 2 years	Control	32.3 ± 0.4 (c)	24.8 ± 0.2 (b)	10.2 ± 0.2 (b)	6.4 ± 0.1 (a)	4.1 ± 0.1 (c)	4.9 ± 0.1 (b)	3.1 ± 0.0 (d)
	ACR1	33.6 ± 0.4 (b)	31.1 ± 0.7 (a)	9.6 ± 0.2 (c)	6.6 ± 0.2 (a)	5.0 ± 0.2 (a)	6.0 ± 0.2 (a)	4.1 ± 0.2 (a)
	ACR5	33.7 ± 0.1 (b)	29.8 ± 0.6 (a)	10.2 ± 0.2 (b)	6.5 ± 0.2 (a)	5.0 ± 0.1 (a)	6.2 ± 0.2 (a)	3.9 ± 0.1 (b)
	35SCR6	34.5 ± 0.1 (a)	26.1 ± 0.9 (b)	11.1 ± 0.4 (a)	6.6 ± 0.2 (a)	4.6 ± 0.1 (b)	5.9 ± 0.1 (a)	3.6 ± 0.1 (c)

Cotton plants were grown at the experimental farm located in Chongqing, China (29°49ʹ11″N, 106°24ʹ43″E). The experiment was designed as a randomized comparative trial with three replicates. Plots were arranged randomly with an area of 18 m^2^ (4 m×4.5 m). Results are presented as means ±SD (*n*=3). Within each column, means that are followed by different letters are significantly different at *P*<0.05 by one-way ANOVA with a Tukey multiple comparisons test. Control, non-transgenic segregated line of *proAGIP::GhCKX3b*-RNAi transgenic cotton; ACR, *proAGIP::GhCKX3b*-RNAi transgenic cotton; 35SCR6, line #6 of *pro35S::GhCKX3b*-RNAi transgenic cotton.

## Discussion

The promotive effect of cytokinins on seed yield was first observed by exogenous application of cytokinins in various crops (Dyer *et al.*, 1987; Atkins and Pigeaire, 1993; Sawan *et al.*, 2000; Zuñiga-Mayo *et al.*, 2018), followed by genetic expression of cytokinin biosynthetic genes, such as the gene for isopentenyltransferase, to endogenously increase the cytokinin levels in transgenic plants (Ma *et al.*, 2008; Atkins *et al.*, 2011; Peleg *et al.*, 2011). CKX, which inactivates cytokinin irreversibly in plant cells, has been identified as a key negative regulator of cytokinin content in monocots and dicots (Jones and Schreiber, 1997; Schmülling *et al.*, 2003; Kowalska *et al.*, 2010; Zeng *et al.*, 2012; Zhao *et al.*, 2015; Ogonowska *et al.*, 2019). Accumulating evidence has demonstrated that CKX is a key regulator for seed number, and down-regulation of CKXs is an effective strategy for yield improvement of seed crops (Zalewski *et al.*, 2010; Bartrina *et al.*, 2011; Li *et al.*, 2013; Yeh *et al.*, 2015; Schwarz *et al.*, 2020). In our previous study, we generated cytokinin-enhanced transgenic cotton in which a *CKX* gene was constitutively down-regulated. We found that some transgenic cottons displayed cytokinin overproduction-related aberrations, including dwarf growth, shortened root, and sterility. Only a moderate increase of cytokinin could show a positive effect on cotton yield (Zhao *et al.*, 2015). In this study, we revealed that *GhCKX3b*, *GhCKX3c*, *GhCKX5a*, and *GhCKX6b* were expressed preferentially in the carpel and stamen (Fig. 1C; Supplementary Fig. S2), suggesting a possible function of these genes in carpel development. We thus designed a *GhCKX3b*-RNAi sequence, which could interfere with the expression of *GhCKX3b*, *GhCKX3c*, *GhCKX5a*, and *GhCKX6b* (Figs 3, 5A; Supplementary Fig. S3; Supplementary Table S5). The cytokinin content in the carpels of transgenic *proAGIP::GhCKX3b*-RNAi cotton lines was significantly higher not only over the wild-type control but also over the positive control *pro35S::GhCKX3b*-RNAi carpels (Table 1; Fig. 4). Compared with *pro35S::GhCKX3b*-RNAi cotton, of which some exhibited abnormal phenotypes, all *proAGIP::GhCKX3b-*RNAi cotton lines grew well without any cytokinin overproduction-related aberrations. More importantly, the fiber yield of the two transgenic *proAGIP::GhCKX3b*-RNAi cotton lines was significantly higher than that of both wild-type control and the *pro35S::GhCKX3b*-RNAi line (Table 2), indicating an advantage of the tissue-specific regulation strategy over the constitutive one.

**Fig. 7.**
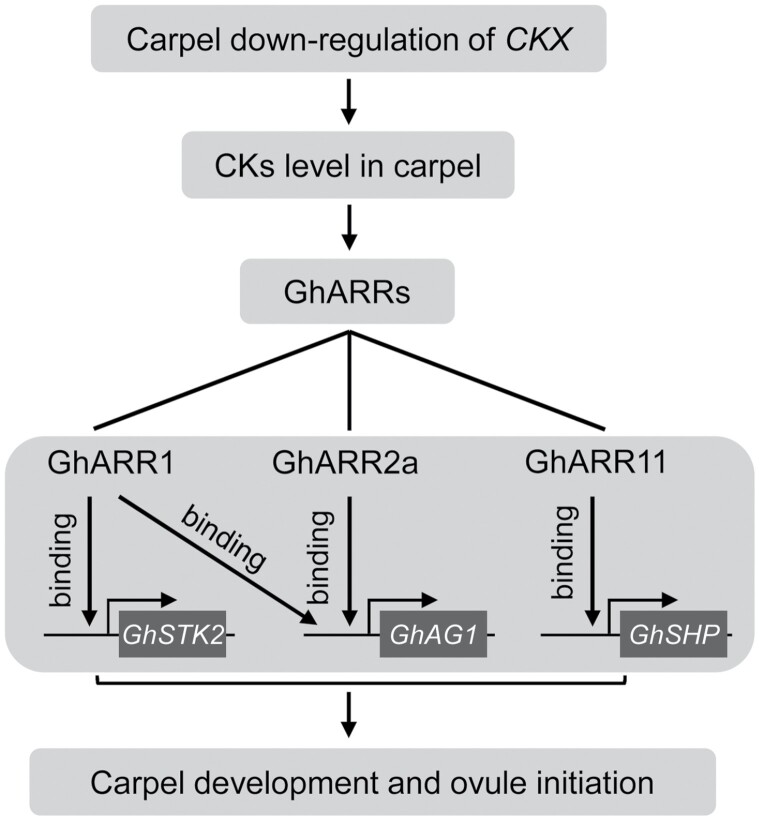
A model showing the promotive effect of carpel-specific enhancement of cytokinins (CKs) on cotton yield. The carpel-specific down-regulation of *GhCKX*s results in an increase of CK levels at the place where ovules initiate. The increased cytokinin content stimulates the activation of B-type ARR (Hwang and Sheen, 2001; Meng *et al.*, 2017), which in turn promotes the transcription of AG subfamily genes (e.g. *GhSTK2*, *GhAG1*, and *GhSHP*) by binding the upstream region of the genes. The up-regulation of these AG subfamily genes boosts carpel differentiation and ovule formation (Favaro *et al.*, 2003; Pinyopich *et al.*, 2003; Xu *et al.*, 2004; Guo *et al.*, 2007; Liu *et al.*, 2009; Liu *et al.*, 2010; de Moura *et al.*, 2017; Nardeli *et al.*, 2018). This promotion of ovule formation results in production of more seeds in a boll, thus increasing the yield of both seeds and fibers of cotton.

Cytokinins regulate growth and development of plants through the cytokinin–ARR signaling pathway (Hwang and Sheen, 2001; Meng *et al.*, 2017). Among ARR-activated genes, AG subfamily genes *STK2*, *AG1*, and *SHP* are positive regulators of ovule identity and initiation (Favaro *et al.*, 2003; Pinyopich *et al.*, 2003; Xu *et al.*, 2004; Guo *et al.*, 2007; Liu *et al.*, 2009, 2010; de Moura *et al.*, 2017; Nardeli *et al.*, 2018). Our transcriptome and RT-qPCR data showed that the expression of AG subfamily genes was obviously increased in the ACR transgenic cotton carpels at the place where ovules formed (Fig. 5A; Supplementary Table S5). Our Y1H and dual-luciferase assays further revealed that GhARR1, GhARR2a, and GhARR11 could directly bind to the upstream sequences of *GhAG1* and *GhSTK2*, *GhAG1*, and *GhSHP*, respectively, and in turn activate the transcription of these AG subfamily genes (Fig. 5C–E; Supplementary Fig. S6). The enhanced expression of these AG subfamily genes could in turn promote carpel development and ovule formation, thus increasing the yield of seeds. The mechanism for increasing cotton yield by the carpel-specific enhancement of cytokinins is summarized in Fig. 7.

Fiber length, strength, and fineness are the three major traits determining the quality and economic value of cotton (Han *et al.*, 2013; Long *et al.*, 2018). It has been reported that a high concentration of kinetin (>5 µM), a type of cytokinin, inhibits fiber elongation, whereas a low concentration (<0.5 µM) stimulates fiber elongation (Beasley and Ting, 1974; Yu *et al.*, 2000b). In addition, fiber elongation was inhibited in transgenic cotton expressing cytokinin biosynthesis isopentenyltransferase gene, *ipt*, under the control of seed-specific promoter Ph/P (Yu *et al.*, 2000a). Our previous study demonstrated that constitutive overexpression of *GhCKX*-RNAi had little negative effect on fiber quality, such as length, strength, and fineness (Zhao *et al.*, 2015). In this current study, we found that the fiber length and fiber strength of the ACR1 transgenic line was significantly decreased, whereas there was no significant alteration in ACR5. We noticed that the seed number per boll of ACR1 (31.1 ± 0.7) was higher than that of ACR5 (29.8 ± 0.6), while its seed size (indicated by the seed index) declined (Table 2). One explanation for the deterioration in quality of transgenic ACR1 fibers is that more seeds per boll means a lower nutritional share, which may reduce the quality of the fibers. The impact of manipulation of cytokinins on fiber quality awaits further investigation.

Taken together, our data indicate that without any hindrance of plant development, carpel-specific up-regulation of endogenous cytokinins by down-regulation of *CKX* is a feasible and effective strategy for seed yield improvement, not only for cotton but perhaps also for other dicotyledons, such as canola and soybean.

## Supplementary data

The following supplementary data are available at *JXB* online.

Fig. S1. Phylogenetic analysis of CKX proteins.

Fig. S2. Transcription levels of *GhCKXs* in different wild-type upland cotton tissues.

Fig. S3. Sequence alignment of partial *GhCKXs* which were preferentially expressed in the carpel.

Fig. S4. The activity pattern of *proAGIP* in *Nicotiana tabacum*.

Fig. S5. Expression patterns and nuclear localization of GhARRs.

Fig. S6. Diagram of ARR binding elements in the AG subfamily gene promoters.

Fig. S7. Comparison of ovule number per 0 DPA locule or boll between T_0_*proAGIP::GhCKX3b*-RNAi transgenic cottons and wild type.

Fig. S8. Comparison of ovule number per 0 DPA locule or boll between T_2_*proAGIP::GhCKX3b*-RNAi transgenic cotton and the non-transgenic segregated line at different growth temperatures.

Fig. S9. Phenotypes of *proAGIP::GhCKX3b*-RNAi transgenic cotton bolls and seeds.

Table S1. Primer and fragment information for plasmid construction.

Table S2. Primers pairs used for RT-qPCR assay.

Table S3. MIQE checklist.

Table S4. Analysis parameters for CKs using LC-MS/MS.

Table S5. Transcriptomic analysis of transcripts that were significantly changed between *proAGIP::GhCKX3b*-RNAi transgenic cotton and the non-transgenic segregated line.

Table S6. Comparison of mature fiber qualities of *proAGIP::GhCKX3b*-RNAi transgenic cottons and the non-transgenic segregated line in field trial.

erac303_suppl_Supplementary_Figures_and_Tables

## Data Availability

The original contributions presented in the study are included in the article and its supplementary data, and further enquiries can be directed to the corresponding author.

## Abbreviations

AG: Agamous
AHK: Arabidopsis histidine kinase receptor
AHP: Arabidopsis histidine protein
ARR: Arabidopsis response regulator
CK: cytokinin
CKX: cytokinin oxidase/dehydrogenase
DPA: days post-anthesis
RT-qPCR: real-time quantitative PCR
SHP: Shatterproof
STK: Seedstick
tZ: *trans*-zeatin
tZR: *trans*-zeatin riboside
