# Supplementary material for: Carpel-specific down-regulation of GhCKXs in cotton significantly enhances seed and fiber yield
Source: J Exp Bot. 2022 Jul 6;73(19):6758–72. doi: 10.1093/jxb/erac303 (PMC9629787; doi:10.1093/jxb/erac303)
Supplement: erac303_suppl_Supplementary_Figures_and_Tables [file erac303_suppl_supplementary_figures_and_tables.pdf]

Supplementary Data

Fig. S1. Phylogenetic analysis of CKX proteins

Fig. S2. Transcription levels of *GhCKXs* in different wild-type upland cotton tissues

Fig. S3. Sequence alignment of partial *GhCKXs* which were preferentially expressed in the carpel

Fig. S4. The activity pattern of *proAGIP* in *Nicotiana tabacum*

Fig. S5. Expression patterns and nuclear localization of GhARRs

Fig. S6. Diagram of ARR binding elements in the AG subfamily gene promoters

Fig. S7. Comparison of ovule number per 0 DPA locule or boll between T_0 *proAGIP::GhCKX3b*-RNAi transgenic cottons and wild type

Fig. S8. Comparison of ovule number per 0 DPA locule or boll between T_2 *proAGIP::GhCKX3b*-RNAi transgenic cotton and the nontransgenic segregated line at different growth temperatures

Fig. S9. Phenotypes of *proAGIP::GhCKX3b*-RNAi transgenic cotton bolls and seeds

Table S1. Primer and fragment information for plasmid construction

Table S2. Primers pairs used for RT-qPCR assay

Table S3. MIQE checklist

Table S4. Analysis parameters for CKs using LC-MS/MS

Table S5. Transcriptomic analysis of transcripts that were significantly changed between *proAGIP::GhCKX3b*-RNAi transgenic cotton and the nontransgenic segregated line

Table S6. Comparison of mature fiber qualities of *proAGIP::GhCKX3b*-RNAi transgenic cottons and the nontransgenic segregated line in field trial

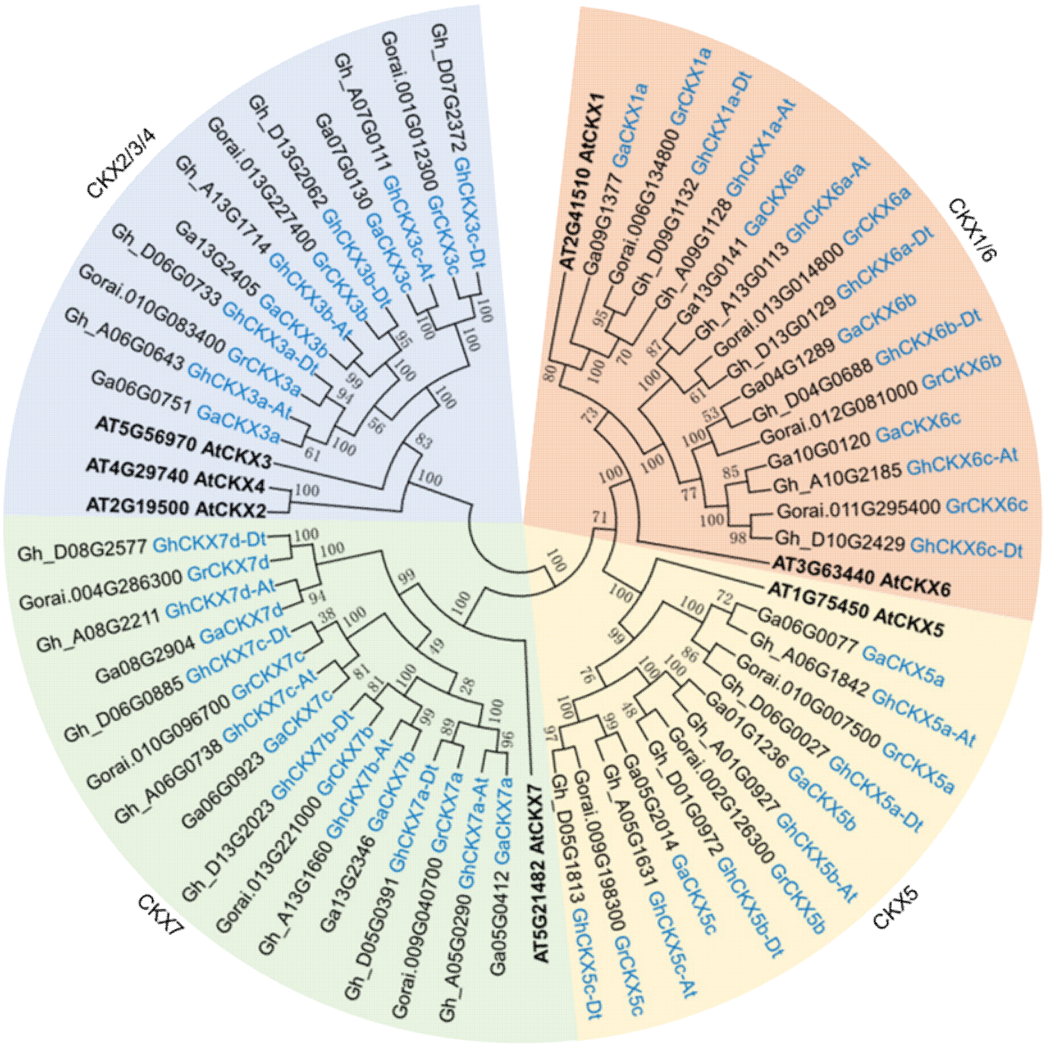

Fig. S1. Phylogenetic analysis of CKX proteins

Amino acid sequences of CKXs in *Arabidopsis thaliana* (At), *Gossypium raimondii* (Gr), *Gossypium arboretum* (Ga), and *Gossypium hirsutum* (Gh) were obtained from https://www.Arabidopsis.org/, https://phytozome.jgi.doe.gov/pz/portal.html, and http://www.cottonfgd.org/, respectively. The suffix "A" and "D" indicates the origin of genes from the cotton A subgenome or D subgenome, respectively. The phylogenetic tree was constructed by the NJ method in MEGA5.0.

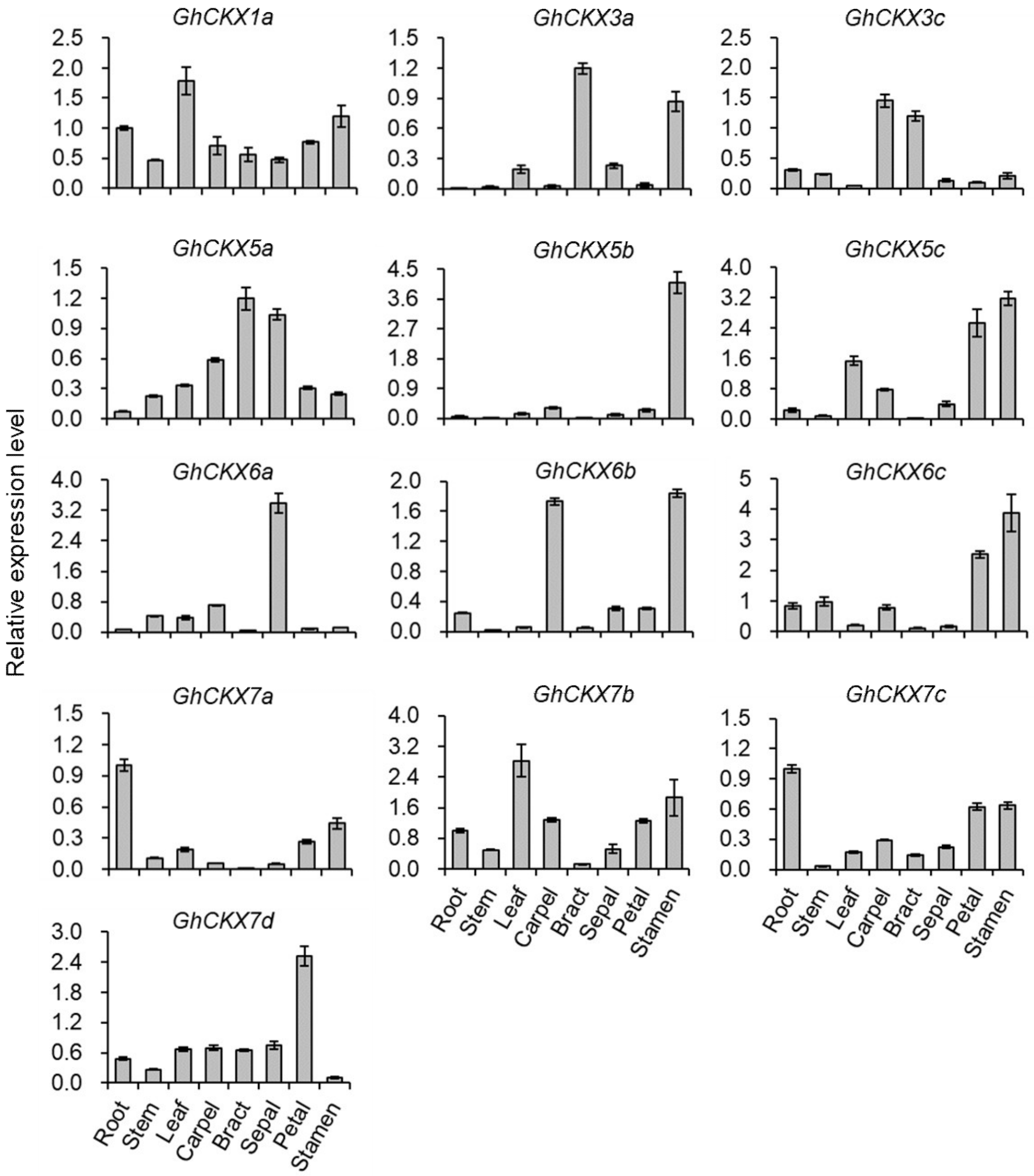

Fig. S2. Transcription levels of *GhCKXs* in different wild type upland cotton tissues *GhCKX* transcription levels detected by RT-qPCR in wild type cotton roots (10 DAS), stems (the third internode at 110 DAS cotton plants), leaves (the third leaf from the apex), carpels, bracts, sepals, petals, and stamens at the pinhead square stage (approximately -21 DPA). *GhHis3* and *GhUbiquitin* served as internal control. Error bars indicate SD of three replicates.

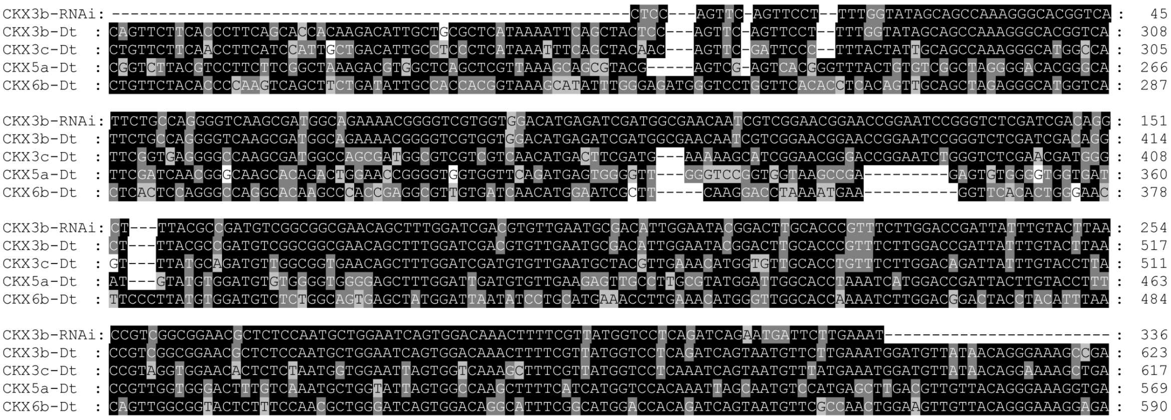

Fig. S3. Sequence alignment of partial *GhCKXs* which are preferentially expressed in the carpel

CKX3b-RNAi, target sequence used for RNA interference (RNAi)-based silencing of *GhCKX3b*. The same background color indicates consistency among the sequences.

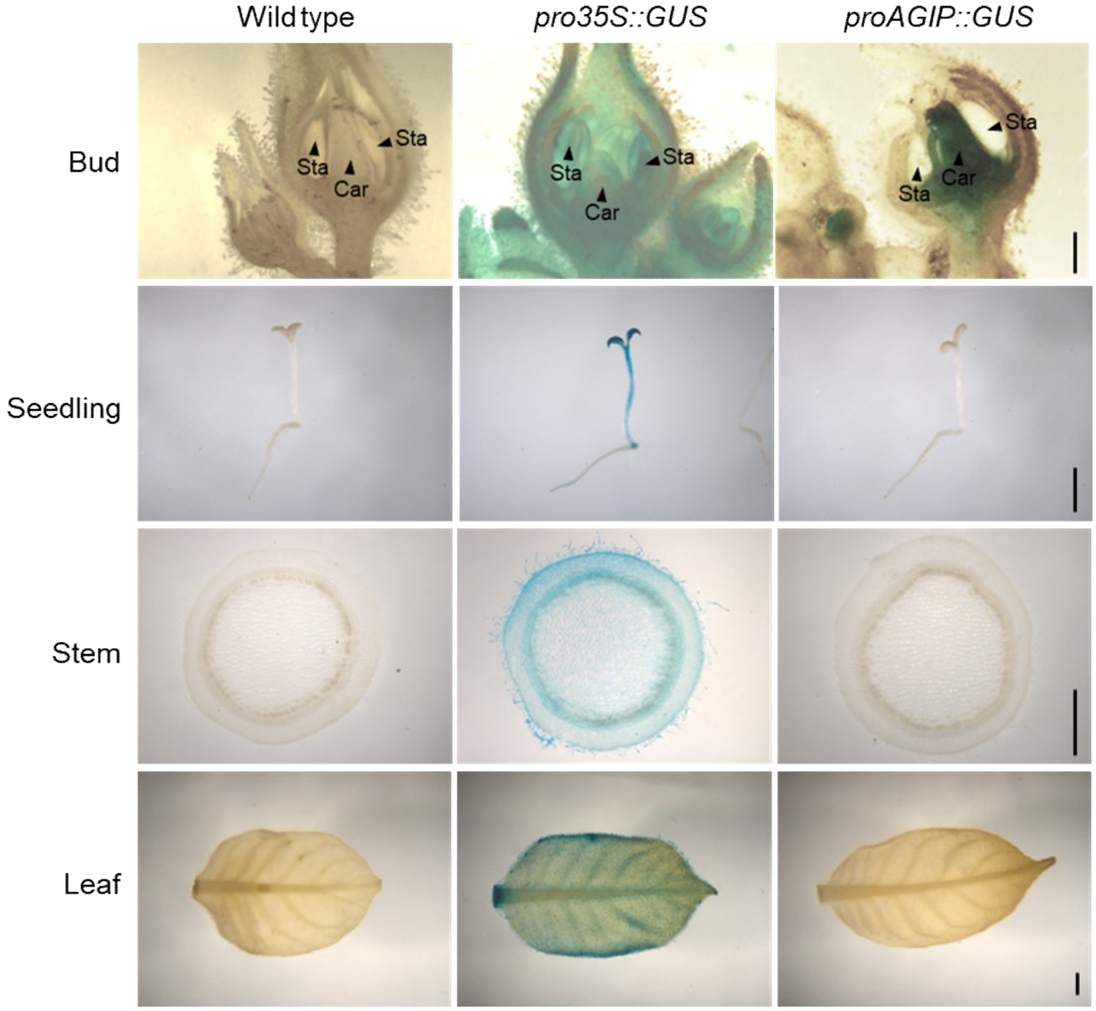

Fig. S4. The activity pattern of *proAGIP* in *Nicotiana tabacum*

The activities of *pro35S* and *proAGIP* were indicated by GUS staining in flower buds (terminal buds at 70 DAS *N. tabacum* plants), seedlings (7 DAS), stems (the first internode from the apex at 70 DAS *N. tabacum* plants) and leaves (the first leaf from the apex). GUS staining of wild type and *pro35S::GUS N. tabacum* tissues served as the negative and positive control, respectively. Representative images are shown based on data from three plants in three independent experiments. Scale bars=2 mm. Car, Carpel; Sta, Stamen.

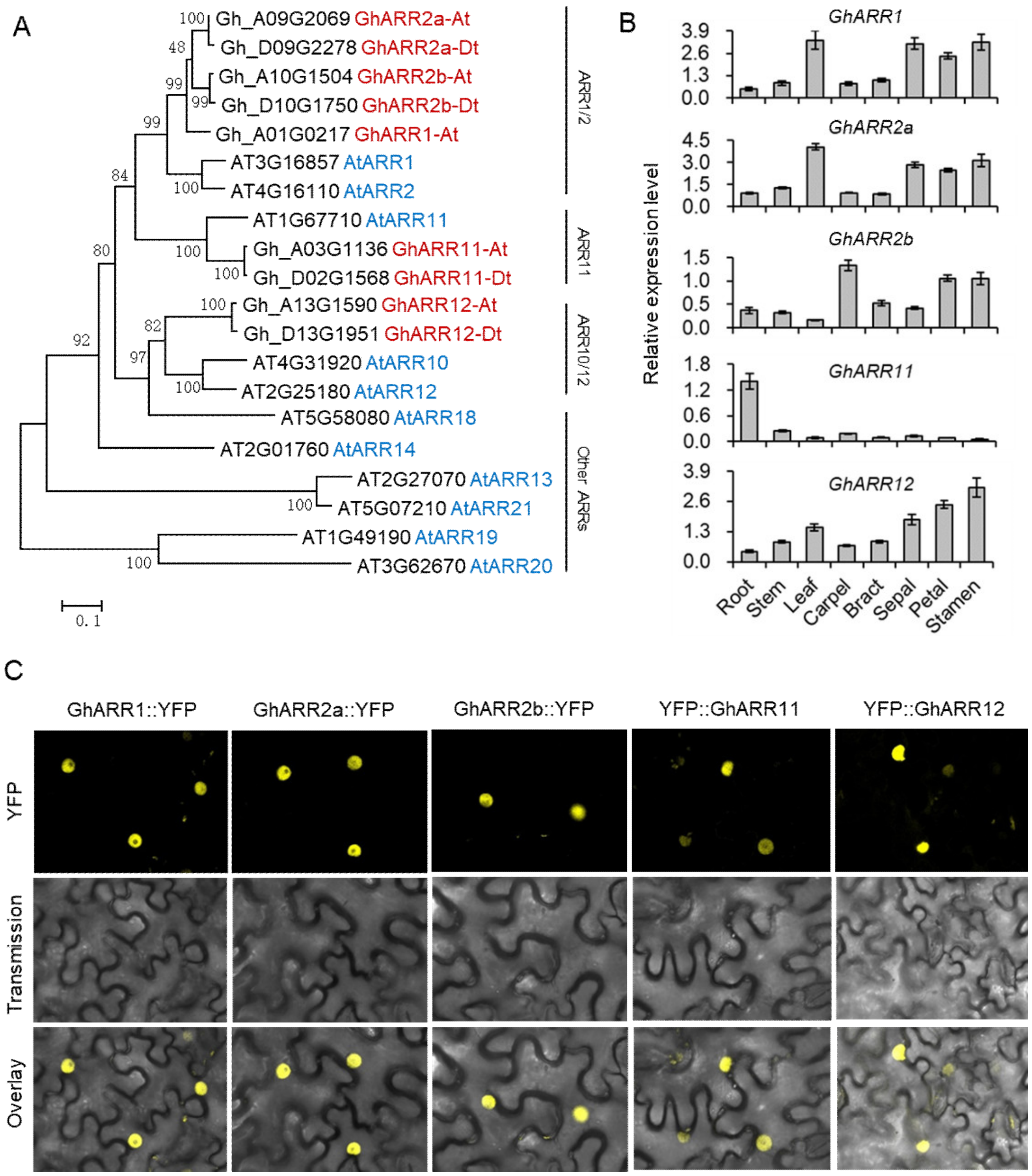

Fig. S5. Expression patterns and nuclear localization of GhARRs

(**A**) Phylogenetic analysis of B-type ARR proteins from *Gossypium hirsutum* and *Arabidopsis thaliana*. The suffix "A" and "D" indicates the origin of genes from cotton A subgenome or D subgenome, respectively. The phylogenetic tree was constructed by the NJ method in MEGA5.0. (**B**) Expression pattern of B-type *GhARRs* in different wild type upland cotton tissues using RT-qPCR analysis. *GhHis3* and *GhUbiquitin* served as internal control. Error bars indicate SD of three replicates. (**C**) Nuclear localization of the GhARR1::YFP, GhARR2a::YFP, GhARR2b::YFP, YFP::GhARR11, and YFP::GhARR12 fusion proteins in *Nicotiana benthamiana* pavement cells.

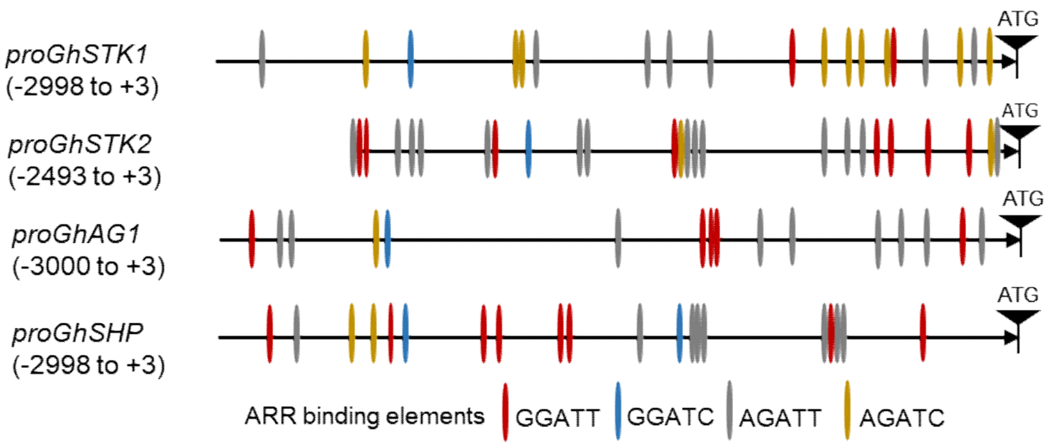

Fig. S6. Diagram of B-type ARR binding elements in the AG subfamily gene promoters

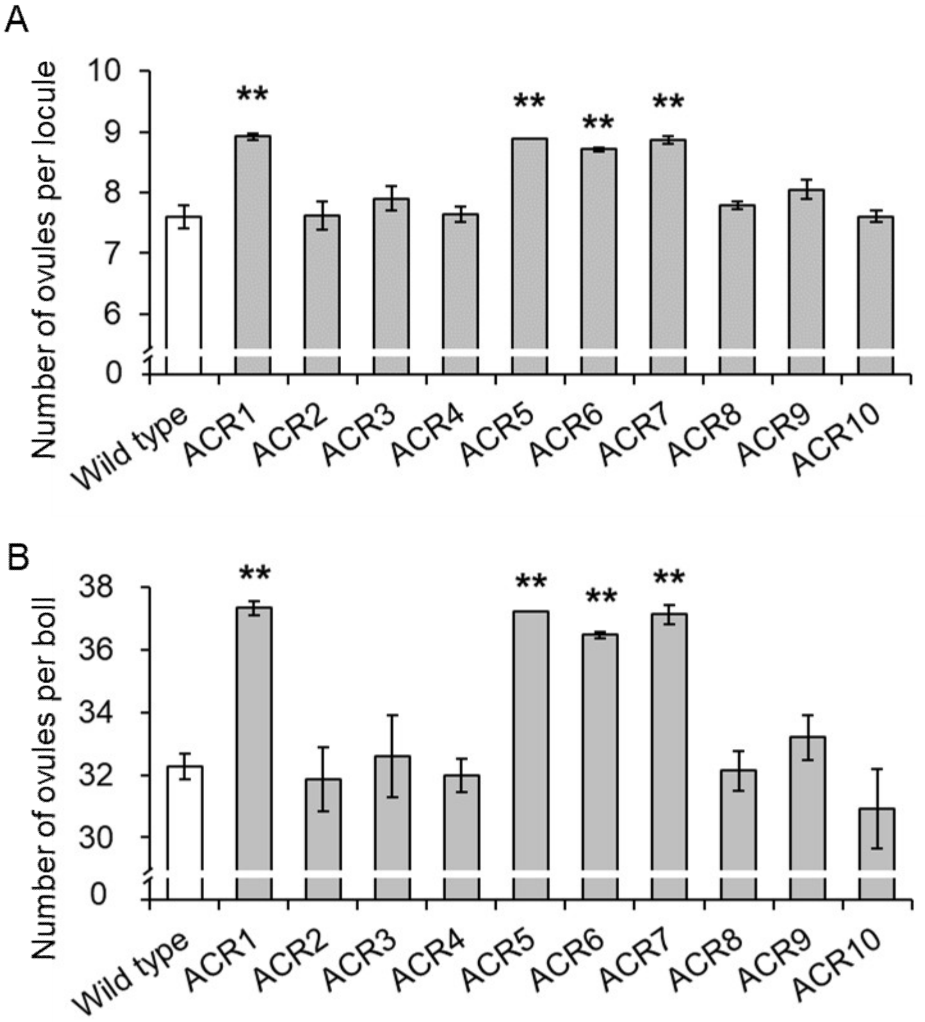

Fig. S7. Comparison of ovule number per 0 DPA locule or boll between T_0 *proAGIP::GhCKX3b*-RNAi transgenic cottons and wild type

Error bars indicate SD of 15 bolls in each experiment. Asterisks represent significant difference (versus wild type) as determined by Student's *t*-test (***P*<0.01).

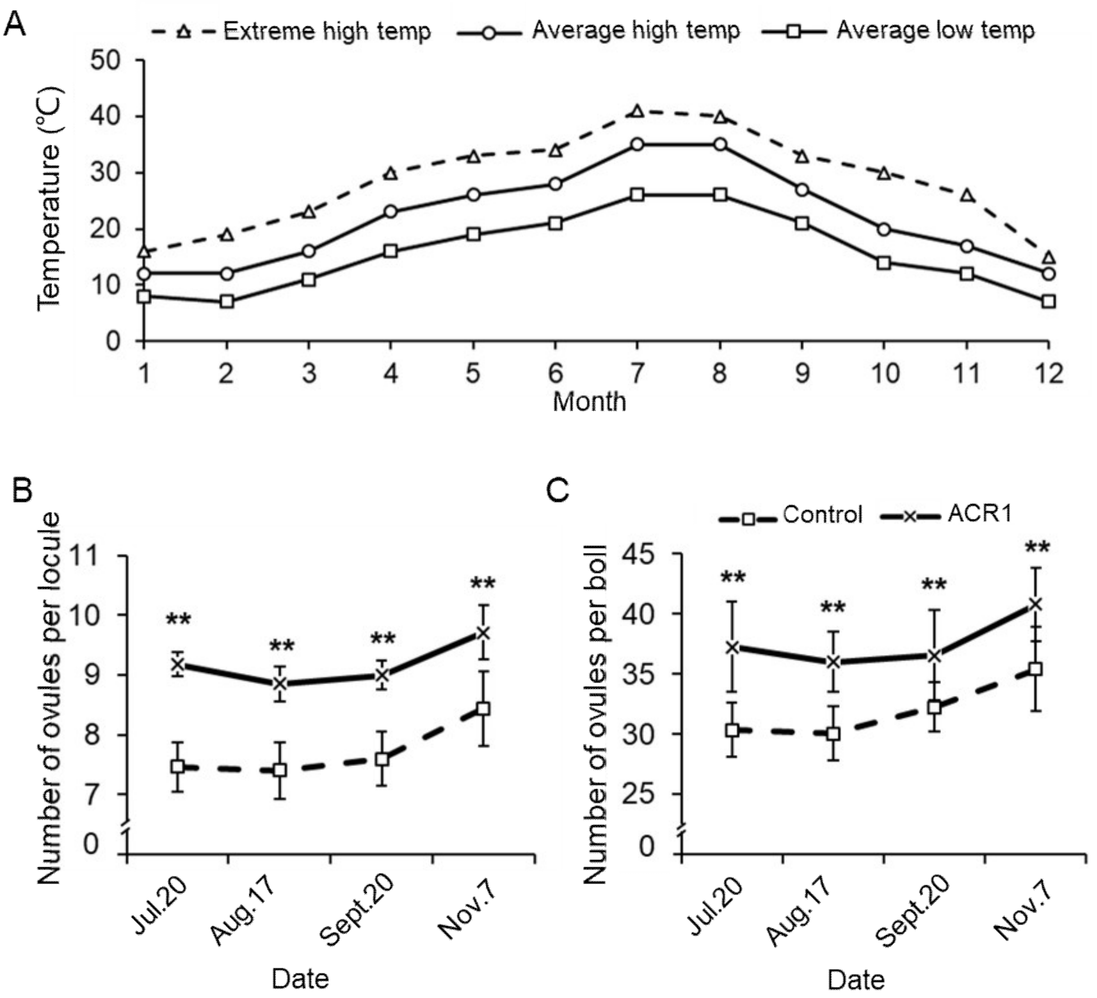

Fig. S8. Comparison of ovule number per 0 DPA locule or boll between T_2 *proAGIP::GhCKX3b*-RNAi transgenic cotton and the nontransgenic segregated line at different growth temperatures

(**A**) Temperature datasets in 2017 were obtained from the website of the China Meteorological Administration (https://www.tianqi.com/). (**B**, **C**) Comparison of ovule number per locule or boll between T_2 *proAGIP::GhCKX3b*-RNAi transgenic cottons and wild type on different dates in 2017. Error bars indicate SD of 15 bolls in each experiment. Asterisks represent a significant difference (versus control), determined by Student's *t*-test (***P*<0.01). Control, nontransgenic segregated line of *proAGIP::GhCKX3b*-RNAi transgenic cotton. ACR, *proAGIP::GhCKX3b*-RNAi transgenic cotton.

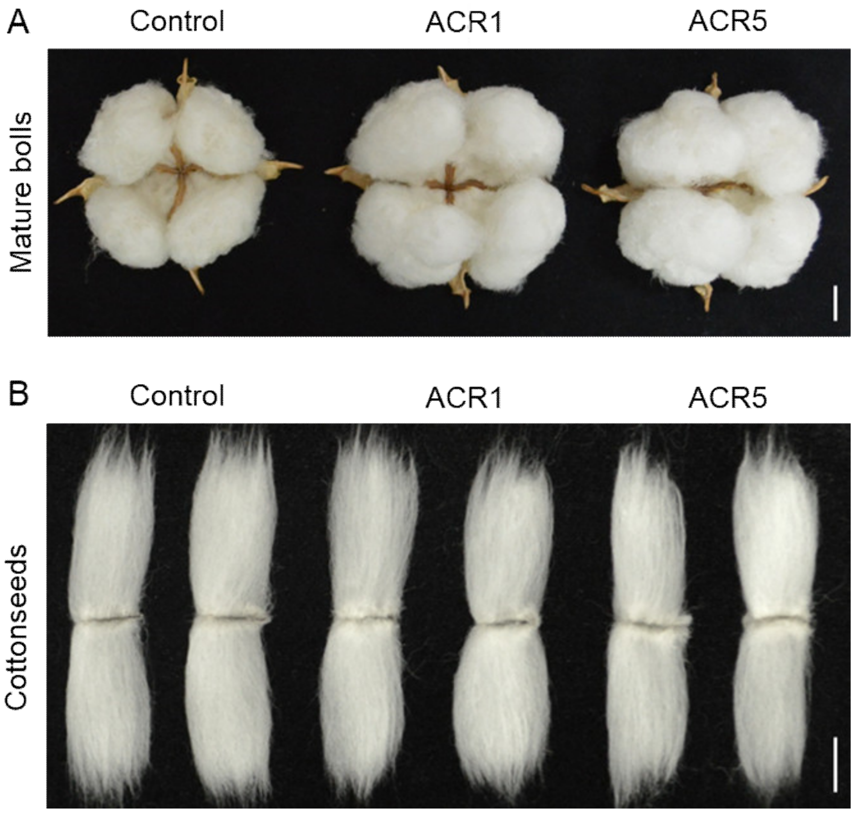

Fig. S9. Phenotypes of *proAGIP::GhCKX3b*-RNAi transgenic cotton bolls and seeds (**A**) Phenotype of mature cotton bolls. (**B**) Comparison of mature fibers on cottonseeds. Scale bars=1 cm. Control, nontransgenic segregated line of *proAGIP::GhCKX3b*-RNAi transgenic cotton. ACR, *proAGIP::GhCKX3b*-RNAi transgenic cotton.

Table S1. Primer and fragment information for plasmid construction

Gene-cassettes	Sequence name	Sequence length (bp)	Primers pairs used for amplification	
			Forward primer (5' > 3')	Reverse primer (5' > 3')
proAGIP::GhCKX3b-RNAi	proAGIP (containing *Hin*dIII and *Sal*I sites)	1720	aagcttcttaatctacgcttaaatctgcat	gtcgacgtgtcctctccaaatgaaatgaacttccttatatagaggaaggg tcggatctctagtaatctctaa
	GhCKX3b-RNAi	1479	gtcgacgctactccagttcagttcct	gaattcggtaccgtaatacgactcac
proAGIP:GUS	proAGIP (containing *Hin*dIII and *Spe*I sites)	1720	aagcttcttaatctacgcttaaatctgcat	actagtgtgtcctctccaaatgaaatgaacttccttatataga
pro35S::GhARR1	GhARR1 (containing *Spe*I and *Sal*I sites, with stop codon)	2049	acggatccgtactagtatgaaaaattcaattggtggaaaag	ctgatcctccgtcgaccactggaatattatccaaggaatac
pro35S::GhARR2a	GhARR2a (containing *Spe*I and *Sal*I sites, with stop codon)	2031	acggatccgtactagtatgaatgtaagtagtgtaaaagg	ctggtaccatgtcgacctacactggaatattattcatag
pro35S::GhARR2b	GhARR2b (containing *Spe*I and *Sal*I sites, with stop codon)	2058	acggatccgtactagtatgaattcaagtagtgggaaatc	ctggtaccatgtcgacttaaactggaatattatcgatgg
pro35S::GhARR11	GhARR11 (containing *Spe*I and *Sal*I sites, with stop codon)	1734	acggatccgtactagtatggtgctcgccgaaagtggctt	ctggtaccatgtcgacttatgcaaacaacccttgatcg
pro35S::GhARR12	GhARR12 (containing *Spe*I and *Sal*I sites, with stop codon)	1854	acggatccgtactagtatgacggtagagcagataggcagtgaaggcaa	ctggtaccatgtcgactcatatggaagtccttagagaataaccgttgcat
proGhSTK1::LUC	proGhSTK1 (containing *Hin*dIII and *Bam*HI sites)	3010	cggtatcgataagcttgggtattggacattgcccaag	tagaactagtggatcctctctttgatcacttgtacctgc
proGhSTK2::LUC	proGhSTK2 (containing *Hin*dIII and *Bam*HI sites)	2505	cggtatcgataagcttccatcatcatatggtaaagactg	tagaactagtggatcccttcaccctttatatattcctac
proGhAG1::LUC	proGhAG1 (containing *Hin*dIII and *Bam*HI sites)	3012	cggtatcgataagcttgatagaacgtgatatgtagaag	tagaactagtggatccgcttcctaactgaaaaaaataaacc
proGhSHP::LUC	proGhSHP (containing *Hin*dIII and *Bam*HI sites)	3010	cggtatcgataagcttgtagaaggatatgagttcgag	tagaactagtggatccggttcaaagctgaaaaaacaagc
pro35S::GhARR1::YFP	GhARR1 (containing *Spe*I and *Sal*I sites, without stop codon)	2046	acggatccgtactagtatgaaaaattcaattggtggaaaag	tgatcctccgtcgaccactggaatattatccaagg
pro35S::GhARR2a::YFP	GhARR2a (containing *Spe*I and *Sal*I sites, without stop codon)	2028	acggatccgtactagtatgaattcaagtagtgggaaatca	tgatcctccgtcgacaactggaatattatcgatgg
pro35S::GhARR2b::YFP	GhARR2b (containing *Spe*I and *Sal*I sites, without stop codon)	2055	acggatccgtactagtatgaatgtaagtagtgtaaaagg	ctgatcctccgtcgaccactggaatattattcatagaatac
	YFP (containing *Sal*I and *Eco*RI sites, and 45 bp linker, with stop codon)	777	gtcgacggaggatcaggaggaggaggatcaggaggaggaggatcaggagg aatggtgagcaagggcgagga	ttaaagcagggaattctcacttgtacagctcgtccatg
pro35S::YFP::GhARR11	GhARR11 (containing *Sal*I and *Eco*RI sites, with stop codon)	1731	tcaggaggagtcgacatggtgctcgccgaaagtgg	ttaaagcagggaattcttatgcaaacaacccttgatcga
pro35S::YFP::GhARR12	GhARR12 (containing *Sal*I and *Eco*RI sites, with stop codon)	1854	gctgtacaaggtcgacatgacggtagagcagatagg	ttaaagcagggaattctcatatggaagtccttagagaat
	YFP (containing *Spe*I and *Sal*I sites, and 60 bp linker, without stop codon)	789	acggatccgtactagtatggtgagcaagggcgagg	gtcgactcctcctgatcctcctcctcctgatcctcctcctcctgatcctcct cctcctgatcctcccttgtacagctcgtccatgc

pGADT7-*GhARR1*	GhARR1 (containing *Nde*I and *Bam*HI sites)	2049	acgacgtaccagattacgctcatatgaaaaattcaattggtggaaaag	atctgcagctcgagctcgatggatccctacactggaatattatccaag
pGADT7-*GhARR2a*	GhARR2a (containing *Bam*HI and *Xho*I sites)	2031	cccgggtgggcatcgatacgggatccatgaattcaagtagtgggaaatc	tatctacgattcatctgcagctcgagttaaactggaatattatcgatg
pGADT7-*GhARR11*	GhARR11 (containing *Eco*RI and *Xho*I sites)	1734	atatggccatggaggccagtgaattcatggtgctcgccgaaag	tatctacgattcatctgcagctcgagttatgcaaacaacc
pAbAi-*proGhSHP*	proGhSHP (containing *Kpn*I and *Xho*I sites)	2487	cttgaattcgagctcggtaccggattaattcgtaaaatcatatattcc	tacagagcacatgcctcgaggcaagctgtatacgtatttatg
pAbAi-*proGhSTK2*	proGhSTK2 (containing *Kpn*I and *Xho*I sites)	1028	aaaagcttgaattcgagctcggtaccattttgtgtgttattattaattaattc	tatacatacagagcacatgcctcgagtctaatcccttatttaaag
pAbAi-*proGhAG1*	proGhAG1 (containing *Hind*III and *Xho*I sites)	2437	aatgatgaattgaaaagcttgaagctaagagatagatcttcaaac	tacagagcacatgcctcgaggcttcctaactgaaaaaaataaacc
GhCKX3b antisense probe	GhCKX3b antisense probe (with an extension)	226	gatgttgggtatgaagagt	*cttctaatacgactcactatag**gg*cataggataaacgaggacag
GhCKX3b sense probe	GhCKX3b sense probe (with an extension)	226	*cttctaatacgactcactatag**gg*gatgttgggtatgaagagt	cataggataaacgaggacag
NPTII (Southern probe)	NPTII	665	cgtaaagcacgaggaag	ctgggcacaacagacaa

The underline indicates the restriction sites. The italic in primer indicates the extension used for the promoter of T7 RNA polymerase.

Table S2. Primers pairs used for RT-qPCR assay

Gene name	Gene ID	Forward primer (5' > 3')	Reverse primer (5' > 3')	Location
GhSTK1	Gh_A05G2136/Gh_D05G2375	gtcgaggccgtctgtatgag	tgctgtctcaacttggctga	Exon1 to Exon3
GhSTK2	Gh_A05G2334/Gh_D05G2596	acaagaaggcatgttccggt	cagctagcagcatttcgtgc	Exon2 to Exon5
GhAG1	Gh_A10G2221/Gh_D10G0308	tccgtagggggattgcctat	acctccgggcatcaaattca	Exon4 to Exon7
GhSHP	Gh_A05G3267/Gh_D04G0341	tgaaggcaggctggagaaag	ccagcaggtttactggcaga	Exon4 to Exon7
GhARR1	Gh_A01G0217	acgtggaaagcaggagatgta	tttccatcatccgccgacat	Exon1 to Exon3
GhARR2a	Gh_A09G2069/Gh_D09G2278	ggttgagtggggtatcgcaa	tgctcacatgttgttgctgc	Exon5
GhARR2b	Gh_A10G1504/Gh_D10G1750	tcatcaatggggcagccaat	aactgggcataaaccctccg	Exon5
GhARR11	Gh_A03G1136/Gh_D02G1568	aagcctttgcttgacgactg	agccatcagtgctggattgt	Exon5
GhARR12	Gh_A13G1590/Gh_D13G1951	tgccatgattcagttgggaca	taaccgttgcatccgccttc	Exon5 to Exon6
GhCKX1a	Gh_A09G1128/Gh_D09G1132	gtttcgggcagtgagttatgg	acaggtaactacttctcctgtacc	Exon1 to Exon2
GhCKX3a	Gh_A06G0643/Gh_D06G0733	agctccaatcccttgttattgc	ctgttccctctggctgctat	Exon1
GhCKX3b	Gh_A13G1714/Gh_D13G2062	ggatgataggatgtcagctgta	ctctggcctggtgacaacaaca	Exon5
GhCKX3c	Gh_A07G0111/Gh_D07G2372	ggcgaacgatggggtcgt	tccaagaaaccggtgcaacac	Exon1
GhCKX5a	Gh_A06G1842/Gh_D06G0027	acctgacaaatcagaaccgtct	caccatgaagccatcttggaa	Exon5
GhCKX5b	Gh_A01G0927/Gh_D01G0972	ccgcatttgggctgatatgg	ccactcctgttgcgtagtgt	Exon5
GhCKX5c	Gh_A05G1631/Gh_D05G1813	agcttctcctcctgggagtt	tagaatggccgtgtcccсta	Exon1 to Exon2
GhCKX6a	Gh_A13G0113/Gh_D13G0129	tcaatcgagacgaaaccgca	tgtttggattttacttcttgggaca	Exon3 to Exon4
GhCKX6b	Gh_D04G0688	ctcggtcagttcggcatcat	gaaatgactggcttgcgctg	Exon2 to Exon3
GhCKX6c	Gh_A10G2185/Gh_D10G2429	agggcattgatgtattgcagga	aacttcccacaagcctctcg	Exon3
GhCKX7a	Gh_A05G0290/Gh_D05G0391	cagtgaccacccaactgtagat	agggtgaggaccatcccaagt	Exon2 to Exon3
GhCKX7b	Gh_A13G1660/Gh_D13G2023	ccgaactgtttttcggagcc	ttgggtcgtcactgttggag	Exon1 to Exon2
GhCKX7c	Gh_A06G0738/Gh_D06G0885	actggctgttcattaccgca	tggacacgaagaggttgagc	Exon2 to Exon3
GhCKX7d	Gh_A08G2211/Gh_D08G2577	ttcatgggttcaaccagggg	gagacttcaacccctccacg	Exon2 to Exon3
GhHis3	Gh_D03G0370	gaagcctcatcgataccgtc	ctaccactaccatcatggc	Exon2 to 3'UTR
GhUbiquitin	Gh_A13G1194/Gh_D13G1489	ccagaaggaatccactttgc	ccagctcacatcagcatacg	Exon1 to 3'UTR

Table S3. MIQE checklist

Item to check	Importance	Details
Experimental design		
Definition of experimental and control groups	E	Mentioned in the manuscript
Number within each group	E	Mentioned in the manuscript
Sample		
Description	E	The vegetative or reproductive organs of cotton
Microdissection or macrodissection	E	Tissues separated according to experimental requirements
Processing procedure	E	Mentioned in the manuscript "Materials and Methods" section
If frozen, how and how quickly	E	Samples were immediately frozen in liquid nitrogen after they were collected
If fixed, with what and how quickly?	E	N/A
Sample storage conditions and duration	E	Samples were held at -80 °C for less than a week before RNA isolation
Nucleic acid extraction		
Procedure and/or instrumentation	E	Total RNA was extracted using the EASY spin plant RNA extraction kit (Aidlab Biotech, China)
Name of kit and details of any modifications	E	EASY spin plant RNA extraction kit (Aidlab Biotech, China). We exactly followed the protocols of the kit
Details of DNase or RNase treatment	E	Genomic DNA was removed by treating with DNase I according to the standard protocols
Contamination assessment (DNA or RNA)	E	No reverse transcription control was performed for each RNA sample to assess the absence of DNA
Nucleic acid quantification	E	RNA concentration was determined by measuring the abosorbance at 260 nm UV light
Instrument and method	E	NanoDrop-2000 Spectrophotometer (Thermo, USA)
RNA integrity: method/instrument	E	RNA integrity was assessed by electrophoresis on 1.0% agarose gels stained with ethidium bromide
RIN/RQI or C_q OF 3' and 5' transcripts	E	N/A
Inhibition testing (C_q dilutions, spike, or other)	E	Standard curve analyses were sufficient to test inhibition
Reverse transcription		
Complete reaction conditions	E	The NovoScript® Plus All-in-one First Strand cDNA Synthesis SuperMix (gDNA Purge, Novoprotein, China) was used to generate first-stranded cDNA. For each sample, a template for no reverse transcription control was prepared
Amount of RNA and reaction	E	Amount of RNA: 1 μg; Reaction volume: 20 μL

volume		
Priming oligonucleotide and concentration	E	oligo-dT: 2 μM
Reverse transcriptase and concentration	E	NovoScript® II Reverse Transcriptase
Temperature and time	E	50 ℃ for 30 min
**qPCR target information**		
Gene symbol	E	Mentioned in the Table S2 of manuscript
Sequence accession number	E	Mentioned in the Table S2 of manuscript
Amplicon length	E	Approximately 200 bp
In silico specificity screen (BLAST, and so on)	E	No test
Location of each primer by exon or intron (if applicable)	E	Mentioned in the Table S2 of manuscript
What splice variants are targeted	E	N/A
**qPCR oligonucleotides**		
Primer sequences	E	Mentioned in the Table S2 of manuscript
Location and identity of any modifications	E	No modifications were done
**qPCR protocol**		
Complete reaction conditions	E	The RT-qPCR assays were performed on a CFX Connect™ Real-Time System (Bio-Rad) with 2×NovoStart®SYBR qPCR SuperMix plus (Novoprotein, China). The PCR reactions were initiated with 3 min incubation at 95 °C, followed by 40 cycles of 95 ℃ for 20 s, 56 ℃ for 20 s, 72 ℃ for 30 s and a standard melting curve to monitor PCR specificity
Reaction volume and amount of cDNA/DNA	E	Reaction volume: 20 μL; amount of cDNA: 50 ng
Primer, (probe), Mg^{2+}, and dNTP concentrations	E	Primer: 200 nM; The Mg^{2+} and dNTP are included in NovoStart®SYBR qPCR SuperMix plus (2×) reagent
Polymerase identity and concentration	E	NovoStart®SYBR qPCR SuperMix plus (2×)
Buffer/kit identity and manufacturer	E	NovoStart®SYBR qPCR SuperMix plus (2×)
Additives (SYBR Green I, DMSO, and so forth)	E	SYBR Green
Complete thermocycling parameters	E	The PCR reactions were initiated with 3 min incubation at 95 °C, followed by 40 cycles of 95 ℃ for 20 s, 56 ℃ for 20 s, 72 ℃ for 30 s and a standard melting curve to monitor PCR specificity.
Manufacturer of qPCR instrument	E	CFX Connect™ Real-Time System (Bio-Rad)
**qPCR validation**		

Specificity (gel, sequence, melt, or digest)	E	Melting curve analysis and gel electrophoresis
For SYBR Green I, C_q of the NTC	E	No signal of the amplification plot (*GhUbiquitin*) was detected or the signal of the amplification plot was very late (*GhUbiquitin*, C_q of NTC > 38).
Calibration curves with slope and y intercept	E	The standard curves were made by real-time quantitative PCR reaction using the gradient dilutions of cDNA or plasmid as the template
PCR efficency calculated from slope	E	95% to 110%
r^2 of calibration curve	E	$r^2 > 0.98$
Linear dynamic range	E	15 to 30 cycles
C_q variation at LOD	E	30 cycles
Evidence for LOD	E	$C_q<35$ for all samples
If multiplex efficiency and LOD of each assay	E	N/A
Data analysis		
qPCR analysis analysis program (sourse, version)	E	CFX Maestro Software
Method of C_q determination	E	C_q values were determined using threshold, which is determined using the Amplification-based Threshold method
Outlier identification and disposition	E	None of C_q values was discarded
Results for NTCs	E	The signal of the amplification plot was very late (C_q>38)
Justification of number and choice of reference genes	E	*GhHis3* and *GhUbiquitin*
Description of normalization method	E	CFX Maestro Software
Number and concordance of biological replicates	D	Triplicate
Number and stage (RT or qPCR) of technical replicates	E	Duplicate
Repeatability (intraassay variation)	E	ΔC_q<0.5 for all duplicates
Statistical methods for results significance	E	No test
Software (source, version)	E	No test

E, essential information; D, desirable information; NTC, no-template control; LOD, limit of detection.

Table S4. Analysis parameters for CKs using LC-MS/MS

Name	Retention time on LC （min）	Q1 > Q3 (Da)	DP (volts)	CE (volts)	CXP (volts)
[$^2H_5$]tZ	14.84	223.0 > 205.0	-82.0	-35.1	-9.1
tZ	15.03	218.0 > 200.0	-82.0	-22.0	-9.8
[$^2H_5$]tZR	20.18	355.1 > 223.0	-48.0	-21.8	-2.0
tZR	20.18	350.2 > 218.0	-140.0	-40.0	-15.0
[$^2H_3$]DZ	15.27	223.0 > 133.0	-87.0	-40.5	-4.1
DZ	15.41	219.9 > 132.9	-87.0	-38.2	-4.7
[$^2H_3$]DZR	20.78	355.1 > 223.1	-49.0	-27.2	-7.0
DZR	20.87	352.0 > 220.1	-49.0	-56.6	-4.0
[$^2H_6$]iP	26.92	208.0 > 133.0	-75.0	-30.2	-10.7
iP	27.23	201.9 > 107.0	-75.0	-24.0	-9.5
[$^2H_6$]iPR	32.23	340.1 > 208.0	-47.0	-26.5	-11.0
iPR	32.51	334.0 > 202.0	-37.0	-19.0	-10.0

DP, declustering potential; CE, collision energy; CXP, collision cell exit potential.

Table S5. Transcriptomic analysis of transcripts that were significantly changed between *proAGIP::GhCKX3b*-RNAi transgenic cotton and the nontransgenic segregated line

Category	Gene name	Gene ID	FPKM						P adjust	Log2FC	Regulated	Significant
			Control-1	Control -2	Control -3	ACR1-1	ACR1-2	ACR1-3				
Cytokinin oxidase	GhCKX3b-At	Gh_A13G1714	0.91	0.90	1.34	0.21	0.23	0.15	3.2E-06	-2.50	down	Yes
	GhCKX3b-Dt	Gh_D13G2062	1.84	2.22	1.47	0.64	1.16	1.08	0.0035	-1.05	down	Yes
	GhCKX3c-At	Gh_A07G0111	1.50	1.50	1.26	0.00	0.00	0.00	7.1E-11	-8.37	down	Yes
	GhCKX5a-At	Gh_A06G1842	3.65	4.24	4.47	0.96	1.80	1.15	7.5E-12	-1.76	down	Yes
	GhCKX5a-Dt	Gh_D06G0027	4.32	4.61	4.90	2.37	2.21	1.68	3.3E-9	-1.24	down	Yes
	GhCKX6b-Dt	Gh_D04G0688	8.73	7.54	7.82	0.05	0.18	0.13	3.8E-43	-6.23	down	Yes
MADS-box genes (AG subfamily)	GhSTK1-At	Gh_A05G2136	4.94	2.47	2.20	17.16	19.25	23.39	1.5E-35	2.22	up	Yes
	GhSTK1-Dt	Gh_D05G2375	4.25	4.23	3.27	17.06	21.21	17.80	9.5E-26	2.11	up	Yes
	GhSTK2-At	Gh_A05G2334	1.86	2.70	2.27	10.66	8.05	7.97	1.1E-9	1.92	up	Yes
	GhSTK2-Dt	Gh_D05G2596	2.55	2.85	2.98	10.43	9.45	10.63	5.9E-16	1.79	up	Yes
	GhSHP-At	Gh_A05G3267	45.19	37.45	38.21	95.64	105.17	102.38	3.9E-53	1.07	up	Yes
	GhSHP-Dt	Gh_D04G0341	53.37	45.34	55.94	117.45	122.08	122.70	6.7E-42	1.06	up	Yes
	GhAG1-At	Gh_A10G2221	18.73	16.59	18.82	39.93	48.02	43.92	5.0E-38	1.13	up	Yes
	GhAG1-Dt	Gh_D10G0308	24.25	19.29	24.86	48.48	59.51	59.28	6.1E-30	1.17	up	Yes

ID, genetic code. log2FC, log2 of fold change. Control, nontransgenic segregated line of *proAGIP::GhCKX3b*-RNAi transgenic cotton. ACR, *proAGIP::GhCKX3b*-RNAi transgenic cotton. Yes, significantly changed DEGs which | log2FC |≥1 and P adjust vaule<0.05 were used as the screening criteria. The suffix "A" and "D" indicates the origin of genes from cotton A subgenome or D subgenome, respectively.

Table S6. Comparison of mature fiber qualities of *proAGIP::GhCKX3b*-RNAi transgenic cottons and the nontransgenic segregated line in field trial

Period	Line	Fiber length (mm)	Fiber strength (cN/tex)	Micronaire
2019 (Jul. 30)	Control	28.5 ± 0.3	30.8 ± 1.2	5.4 ± 0.2
	ACR1	26.9 ± 0.3*	26.9 ± 0.2*	5.1 ± 0.1**
	ACR5	28.1 ± 0.3	30.9 ± 1.2	4.7 ± 0.1*
2019 (Aug.20)	Control	28.0 ± 0.4	29.2 ± 0.2	5.3 ± 0.2
	ACR1	27.1 ± 0.5	27.7 ± 0.4**	5.4 ± 0.2
	ACR5	28.6 ± 0.7	31.0 ± 0.8	4.9 ± 0.2
2019 (Sept.15)	Control	28.5 ± 0.3	33.4 ± 2.4	5.4 ± 0.2
	ACR1	27.6 ± 0.3*	28.3 ± 0.7	5.3 ± 0.2
	ACR5	28.7 ± 0.6	29.9 ± 0.7	4.8 ± 0.1*
Average over 3 periods	Control	28.4 ± 0.4	31.1 ± 2.3	5.3 ± 0.2
	ACR1	27.2 ± 0.4**	27.5 ± 0.7**	5.3 ± 0.2
	ACR5	28.5 ± 0.5	30.6 ± 0.9	4.8 ± 0.1**

Significant differences compared with wild type were determined using Student's *t*-test (*P<0.05; **P<0.01). Control, nontransgenic segregated line of *proAGIP::GhCKX3b*-RNAi transgenic cotton. ACR, *proAGIP::GhCKX3b*-RNAi transgenic cotton.
